# Chronic exposure to IL-6 induces a desensitized phenotype of the microglia

**DOI:** 10.1186/s12974-020-02063-1

**Published:** 2021-01-22

**Authors:** Mireia Recasens, Beatriz Almolda, Jeús Pérez-Clausell, Iain L. Campbell, Berta González, Bernardo Castellano

**Affiliations:** 1 Department of Cell Biology, Physiology and Immunology, Institute of Neuroscience, Universitat Autònoma de Barcelona, 08193, Bellaterra, Barcelona, Spain; 2 Department of Cell Biology, Physiology and Immunology, Universitat de Barcelona, 08028 Barcelona, Spain; 3 School of Life and Environmental Sciences and Charles Perkins Centre, University of Sydney, Sydney, NSW 2006 Australia

**Keywords:** Axonal sprouting, Primed microglia, T cell, Monocyte, MHCII

## Abstract

**Background:**

When the homeostasis of the central nervous system (CNS) is altered, microglial cells become activated displaying a wide range of phenotypes that depend on the specific site, the nature of the activator, and particularly the microenvironment generated by the lesion. Cytokines are important signals involved in the modulation of the molecular microenvironment and hence play a pivotal role in orchestrating microglial activation. Among them, interleukin-6 (IL-6) is a pleiotropic cytokine described in a wide range of pathological conditions as a potent inducer and modulator of microglial activation, but with contradictory results regarding its detrimental or beneficial functions. The objective of the present study was to evaluate the effects of chronic IL-6 production on the immune response associated with CNS-axonal anterograde degeneration.

**Methods:**

The perforant pathway transection (PPT) paradigm was used in transgenic mice with astrocyte-targeted IL6-production (GFAP-IL6Tg). At 2, 3, 7, 14, and 21 days post-lesion, the hippocampal areas were processed for immunohistochemistry, flow cytometry, and protein microarray.

**Results:**

An increase in the microglia/macrophage density was observed in GFAP-IL6Tg animals in non-lesion conditions and at later time-points after PPT, associated with higher microglial proliferation and a major monocyte/macrophage cell infiltration. Besides, in homeostasis, GFAP-IL6Tg showed an environment usually linked with an innate immune response, with more perivascular CD11b^+^/CD45^high^/MHCII^+^/CD86^+^ macrophages, higher T cell infiltration, and higher IL-10, IL-13, IL-17, and IL-6 production. After PPT, WT animals show a change in microglia phenotype expressing MHCII and co-stimulatory molecules, whereas transgenic mice lack this shift. This lack of response in the GFAP-IL6Tg was associated with lower axonal sprouting.

**Conclusions:**

Chronic exposure to IL-6 induces a desensitized phenotype of the microglia.

**Supplementary Information:**

The online version contains supplementary material available at 10.1186/s12974-020-02063-1.

## Background

It is well established that perturbation in the central nervous system (CNS) as a consequence of an acute or chronic injury leads to microglial cell activation. This activation is totally dependent on the type, location, and duration of damage as well as the specific environment in which microglial cell activation takes place [45, 61]. Recent progress in cell-specific transcriptome profiling has been contributed in deciphering the role of microglia in both physiological and pathological conditions, revealing microglia acquire different cell activation signatures associated with an acute injury, chronic neurodegenerative diseases, or aging [48]. Among the different factors controlling microglial cell activation, cytokines and especially the balance between pro- and anti-inflammatory molecules play an essential role [46].

Among the plethora of cytokines that can act on microglia, IL-6 is a molecule that plays an important function as a regulator of inflammatory and immunological responses not only in the periphery [54, 55, 89] but also in the CNS. In the healthy CNS, neurons, glial cells, and endothelial cells express low levels of IL-6 and IL-6R [23, 34, 79, 83, 84]. However, an upregulation of these two molecules is observed after acute insults, such as traumatic brain injury and ischemia, as well as in chronic neurodegenerative conditions like Alzheimer’s and Parkinson’s diseases [11, 13, 30, 71, 86]. Both detrimental and beneficial functions have been described for IL-6 [19, 77]. On the one hand, IL-6 has been classified as a pro-inflammatory cytokine with a noxious role, based on the evidence that, together with other cytokines like TNF-α and IL-1β, acts as a major stimulator of the inflammatory response in the CNS [11, 30, 35], promoting neuronal death [3, 22]. Nevertheless, other studies indicated that IL-6 might have anti-inflammatory and neuroprotective effects after different kind of injuries including spinal cord injury [20], ischemia [64], and sciatic nerve transection [51]. Concerning microglia, in vitro and in vivo studies showed the capacity of IL-6 to induce activation, increasing microglial cell proliferation, as well as the production of microglia-derived pro-inflammatory molecules [15, 17, 21, 57, 91]. Moreover, IL-6 also can drive lymphocyte differentiation as demonstrated by some studies where, in the presence of TGF-b, T cell responses are polarized toward a T-regulatory phenotype, whereas the addition of IL-6 changes the phenotype of T lymphocytes toward a Th17 pathogenic phenotype [12].

The objective of the present study was to investigate the effects of chronic exposure to IL-6 on the phenotype of microglia cells, linked to anterograde axonal degeneration. For this purpose, perforant pathway transection were performed in the GFAP-IL6 transgenic (Tg) mouse model, in which chronic production of IL-6 was selectively targeted to astrocytes in the CNS [16, 17]. This type of lesion produces a dense anterograde and terminal axonal degeneration, glial cell activation, and axonal sprouting within the molecular layer of the fascia dentata, a very specific area of the hippocampus, making it a very useful tool to study modifications in these responses associated to variations in the microenvironment. Hence, the PPT paradigm has been extensively used as a model to study the mechanisms that initiate the innate immune response and the microglial response without blood-brain barrier disruption [6, 32].

## Materials and methods

### Animals

For the present study, a total of 94 GFAP-IL6Tg animals [17] (6–7 months old) and 79 wild-type (WT) littermates of both sexes were used. Animals were maintained at constant temperature (24 ± 2 °C) and housed on a 12-h light/dark cycle with food and water ad libitum during all the experiment. All experimental animal work was conducted in accordance to Spanish regulations (Ley 32/2007, Real Decreto 1201/2005, Ley 9/2003, and Real Decreto 178/2004) in agreement with European Union directives (86/609/CEE, 91/628/CEE, and 92/65/CEE) and was approved by the Ethical Commission of the Autonomous University of Barcelona. All efforts were made to minimize the number of animals used to produce reliable scientific data, as well as animal suffering.

### Perforant pathway transection and experimental groups

GFAP-IL6Tg (*n* = 76) and WT mice (*n* = 62) were subjected to wire-knife unilateral perforant pathway transection (PPT). Briefly, animals were anaesthetized with intraperitoneal injection of a solution of ketamine (80 mg/kg) and xylazine (20 mg/kg) at dose of 0.01 mL/g body weight. Anaesthetized mice were placed in a stereotaxic device (Kopf Instruments®) and a small window in the skull was created by drilling in the left side of the skull (4.6 mm dorsal to Bregma and 2.5 mm laterally). A folded wire-knife (McHugh Milleux, m121) was inserted at an angle of 15° anterior and 10° lateral. The knife was unfolded at 3.6 mm ventrally and the perforant pathway (PP) was transected retracting the knife 3.3 mm. Finally, the knife was folded and removed from the brain. After surgery, the skin was sutured with 2-0 silk and the wound cleaned with iodine.

Non-lesioned (NL) and lesioned animals were distributed in different experimental groups for immunohistochemistry (IHC), flow cytometry, and protein analysis, as detailed in Table 1.

**Table 1:** Experimental groups of animals

		NL	2dpl	3dpl	7dpl	14dpl	21dpl
WT	IHC (*n* = 45)	6	6	8	10	8	7
	FC (*n* = 22)	8		7	7		
	Protein (*n* = 12)	3		3	3	3	
GFAP-IL6Tg	IHC (*n* = 53)	7	7	9	9	10	11
	FC (*n* = 24)	8		8	8		
	Protein (*n* = 17)	3		5	4	5	

### 5′ Bromodeoxyuridine injections

In order to determine microglia/macrophage proliferation, the labeling of proliferative cells with 5′ bromodeoxyuridine (BrdU) was used. BrdU is a synthetic thymidine analog that incorporates into the DNA of dividing cells during S-phase and can be transferred to daughter cells upon replication. Lesioned WT (*n* = 5) and GFAP-IL6Tg animals (*n* = 6) were intraperitoneally (i.p.) injected with BrdU (50 mg/kg) diluted in TB (0.05 Trizma base, pH 7.4) every 24 h from the day of lesion to 7 days post-lesion (dpl), and subsequently euthanized at 7dpl.

### Tissue processing for histological analysis

Animals were anaesthetized as described above, but at 0.015ml/g body weight concentration, and then perfused intracardially for 10 min with 4% paraformaldehyde in 0.1 M phosphate buffer (pH 7.4). Brains were immediately removed and post-fixed for 4 h at 4 °C in the same fixative and, after phosphate buffer rinses, cryopreserved in a 30% sucrose solution in 0.1 M phosphate buffer for 48 h at 4 °C and frozen in ice-cold 2-methylbutane solution (320404, Sigma-Aldrich). A series of horizontal parallel sections (30-μm-thick) were obtained using a Leica CM3050 cryostat and stored free-floating in Olmos anti-freeze solution at -20°C until used.

### Toluidine blue staining

Sections were mounted onto gelatinized slides, air dried at RT for 1 h, and then were incubated for 1 min in a solution containing 0.1% toluidine blue diluted in Walpole’s buffer (0.05 M, pH 4.5). After washes in distilled water, sections were dehydrated in graded alcohols, *N*-butyl alcohol, and after xylene treatment, coverslipped with DPX mounting media.

### Single stain immunohistochemistry

Free-floating sections were processed for the study of microglial morphology, distribution, density, and phenotype using antibodies against Iba1, Pu.1, CD45, MHCII, CD206 (Table 2). For the analysis of microglia proliferation, sections were stained with anti-phospho-histone H3 (pH3) and BrdU, whereas for microglial cell death evaluation, anti-caspase 3 antibody was used. Finally, lymphocyte recruitment was analyzed using antibody against CD3, a pan marker for T lymphocytes. In all cases, sections were washed several times with 0.05 M Tris-buffered saline (TBS) pH 7.4 and with TBS containing 1% Triton-X100 (TBST, pH 7.4). In the case of Pu.1 staining, sections were exposed to antigen retrieval by treatment with sodium citrate buffer (pH 8.5) for 40 min at 80 °C. Endogenous peroxidase was blocked by incubating the sections for 10 min with 2% H_2_O_2_ in 70% methanol. For BrdU detection, DNA was denatured by first incubating in 0.082 N HCl for 10 min at 4 °C and then for 30 min in 0.82 N HCl at 37 °C. Sections were then rinsed with borate buffer (pH 8.5) and 0.5% Triton X-100 in TBS. Afterwards, all sections were incubated for 1 h at room temperature (RT) in blocking buffer solution (BB) containing 10% fetal bovine serum in TBST. After that, sections were incubated overnight at 4 °C followed by 1h at RT with primary antibodies diluted in BB, as specified in Table 2. Sections from spleen and gut were used as positive control whereas sections incubated in BB lacking the primary antibody were used as negative control. After washes with TBST, sections were incubated for 1 h at RT with biotinylated anti-rabbit secondary antibody biotinylated anti-hamster secondary antibody or biotinylated anti-rat secondary antibody (Table 2) diluted in BB. After 1 h at RT in streptavidin-peroxidase, the reaction was visualized by incubating the sections in the 3,3-diaminobenzidine (DAB) kit (SK-4100; Vector Laboratories, USA) following the manufacturer’s instructions. Finally, sections were mounted on slides, counterstained with 1% toluidine blue, dehydrated in graded alcohols, and, after xylene treatment, coverslipped with DPX. Sections were analyzed with a Nikon Eclipse 80i brightfield microscope and photographed with a DXM 1200F Nikon digital camera.

**Table 2:** Reagents used for IHC

	Target antigen	Host	Dilution	Cat Number	Manufacturer
Primary antibodies	Iba1	Rabbit	1:3000	019-19741	Wako
	Pu.1	Rabbit	1:400	2258S	Cell Signaling
	CD45	Rat	1:1000	MCA1031G	AbD Serotec
	pH3	Rabbit	1:3000	06-570	Millipore
	BrdU	Rat	1:120	Ab6326	Abcam
	Caspase 3	Rabbit	1:1000	AF835	R&D Systems
	Iba1	Rabbit	1:1000	GTX100042	Genetex
	CD3	Hamster	1:500	MCA2690	AbD Serotec
	MHCII (IA)	Rat hybridoma	1:25	TIB-120	ATCC
	CD206	Rat	1:500	MCA2235GA	AbD Serotec
	CD28	Rabbit	1:50	ab203084	Abcam
	CTLA-4	Rabbit	1:25	ab237712	Abcam
	Laminin	Rabbit	1:500	AMP420	Biorad
	TMEM119	Rabbit	1:1000	ab209064	Abcam
	GFAP	Mouse	1:3000	63893	Sigma
Secondary antibodies	Biotinylated	Rabbit	1:500	BA-1000	Vector Laboratories
	Biotinylated	Hamster	1:500	BA-9100	Vector Laboratories
	Biotinylated	Rat	1:500	BA-4001	Vector Laboratories
	Alexa 555	Rat	1:1000	A11006	Invitrogen
	Alexa 488	Rabbit	1:500	A21206	Invitrogen
	Cy2	Mouse	1:500	PA42003	GE Healthcare Lifescience
Streptavidin Alexa Fluor-555			1:500	S32355	Molecular Probes
Streptavidin Alexa Fluor-488			1:500	S11223	Molecular Probes
Streptavidin-HRP			1:500	SA-5004	Vector Laboratories
DAPI			1:10000	D9542	Sigma Aldrich

### Double stain immunohistochemistry

Double immunolabeling for BrdU with either Iba1 or GFAP was carried out to identify proliferating microglia/macrophages and astrocytes. Sections were firstly processed for BrdU as described above, with anti-rat Alexa-Fluor 555-conjugated antibody as secondary antibody (Table 2). After several washes with TBST and 1 h incubation in BB at RT, sections were incubated overnight at 4 °C followed by 1 h at RT with rabbit anti-Iba1 and mouse anti-GFAP antibody diluted in BB (Table 2). After washes in TBST, sections were incubated with either anti-rabbit Alexa-Fluor 488-conjugated or anti-mouse Cy2-conjugated secondary antibodies (Table 2). Finally, sections were washed with TBST, followed by TBS and TB, and the nuclei stained with 4,9,6-diamidino-2-phenylindole (DAPI) for 10 min (Table 2).

To study the expression of co-stimulatory molecules on T cells double immunolabeling combining CD28 or CTLA-4 with CD3 were performed (Table 2). Sections were firstly processed for CD3 as described above, but using Alexa-Fluor 488-conjugated streptavidin instead of horseradish streptavidin-peroxidase (Table 2). Then, sections were incubated overnight at 4 °C and 1 h at RT with anti-CD28 and anti-CTLA-4 followed by anti-rabbit Alexa-Fluor 555-conjugated secondary antibody (Table 2).

To analyze the localization of MHCII^+^ cells, double immunofluorescence combining MHCII with laminin was performed (Table 2). Sections were firstly processed for MHCII as described above, but using Alexa-Fluor 555-conjugated streptavidin instead of horseradish streptavidin-peroxidase (Table 2). Then, sections were incubated overnight at 4 °C and 1 h at RT with anti-laminin followed by anti-rabbit Alexa-Fluor 488-conjugated secondary antibody (Table 2).

To study the phenotype of MHCII^+^ cells, double immunofluorescence combining MHCII with Tmem119, a defined microglia-specific marker [10], was performed (Table 2). Sections were firstly processed for Tmem119 as described above, but using Alexa-Fluor 488-conjugated streptavidin instead of horseradish streptavidin-peroxidase (Table 2). After that, sections were incubated overnight at 4 °C and 1 h at RT with anti-MHCII followed by anti-rat Alexa-Fluor 555-conjugated secondary antibody (Table 2).

Sections incubated in BB lacking the primary antibody were used as negative control and sections from spleen and spinal cord from EAE-induced mice were used as positive control for the different immunostains. Finally, all double-immunostained sections were mounted on slides, coverslipped with Fluoromount GTM (0100-01; SouthernBiotech, Birmingham, AL), and analyzed using Nikon Eclipse E600 fluorescence microscope and a Zeiss LSM 700 confocal microscope using a × 40 objective.

### Terminal dUTP nick end labeling

In addition to caspase-3 immunohistochemistry, the study of microglial cell death was performed on sections double stained for terminal dUTP nick end labeling (TUNEL) and Iba1. Briefly, sections were mounted on slides and treated for 5 min with 100% methanol for endogenous peroxidase blocking. Then, sections were rinsed in 10 mM Tris buffer (pH 8) and 5 mM EDTA followed by incubation for 15 min at RT in the same buffer plus Proteinase K (20 μg/mL). After several washes with 5 mM EDTA, sections were incubated for 10 min at RT in TdT buffer containing 30 mM Tris, 140 mM sodium cacodilate, and 1 mM cobalt chloride (pH 7.7). After that, sections were incubated for 20 min at 37 °C in TdT buffer plus 0.161 U/μL TdT enzyme (Terminal Transferase, 3333566 Roche, Manheim, Germany) and 0.0161 nmol/μL of biotin-16-dUTP (1093070, Roche, Manheim, Germany). The reaction was stopped by submerging sections twice in citrate buffer (300 mM sodium chloride, 30 mM sodium citrate, 5 mM EDTA) for 5 min. After several washes with TBS, sections were incubated for 1 h at RT with HRP-conjugated streptavidin (Table 2) and the peroxidase reaction visualized by incubation in a 3,3-DAB kit plus 1% of cobalt (SK-4100; Vector Laboratories, USA), following the manufacturer’s instructions. After that, sections were incubated with Iba1 antibody (1:1000) (Table 2) using the same protocol described in the single IHC section. Sections from gut were used as positive control. Finally, sections were dehydrated in graded alcohols and, after xylene treatment, coverslipped in DPX.

### Sulfide-silver staining

Collateral sprouting in non-lesioned and lesioned animals was analyzed at 14 and 21 dpl, using a variation of the sulfide-silver staining technique, as described by Danscher [24]. Briefly, animals were injected intraperitoneally with sodium selenite (10 mg/kg) and 1 h later, animals were perfused and postfixed as described above. After that, brains were removed and cryoprotected with 30% sucrose solution in 0.1 M phosphate buffer at 4 °C for 48 h and frozen as described above. Frozen parallel transversal sections (30 μm thick) were obtained using a cryostat (Leica, CM 3050S) and mounted on slides. Sections were incubated to visualize metal precipitates. Briefly, slides were rinsed in 95% EtOH for 15 min, then 2 min in 70% alcohol, 50% alcohol, and rinsed again with dH_2_O for 30 min. Following rehydration, sections were incubated with the developer solution that contains 60 mL of arabic gum (0.5 kg/L), 10 mL of sodium citrate buffer 0.2 M, 15 mL hydroquinone (0.85 g diluted in 15 mL of dH_2_O), and 15 mL silver lactate (0.12 g diluted in 15 mL of dH_2_O) in a water bath at 26 °C protected from light for 60–80 min [24]. After that, sections were rinsed in 5% sodium thiosulfate for 12 min and rinsed again with dH_2_O. Sections were postfixated in 70% alcohol for 30 min, dehydrated in alcohol, rinsed in xylene and coverslipped with DPX.

### Brightfield microscopy densitometric analysis

Densitometric analysis in NL and lesioned animals was performed on sections immunolabeled for Iba1 to assess microglia state. A minimum of three animals per genotype and experimental time-point were analyzed. A total of 9 photographs from 3 different sections per animal containing the left molecular layer (ML) of the dentate gyrus (DG) were captured using the × 20 objective with a DXM 1200F Nikon digital camera joined to a brightfield Nikon Eclipse 80i microscope, using the software ACT-1 2.20 (Nikon corporation) (Suppl. Fig. 1). By means of analySIS® software, both the percentage of area occupied by the immunolabeling as well as the intensity of the immunostain (Mean Grey Value Mean) was recorded for each photograph. The AI index [2] was calculated as function of the percentage of the immunostained area and the Mean Grey Value Mean.

In order to quantify microglial cell density, sections immunostained for the transcription factor Pu.1 from a minimum of three NL and three lesioned WT and GFAP-IL6Tg at 2, 3, 7, 14, and 21 dpl were analyzed. In this case, a total of 6 photographs from 3 different sections per animal were captured with the × 10 objective, using the same device and software referred above. The number of Pu.1^+^ cells in the ML of the DG was obtained using the “Automatic Cell Counter” (ITCN) plug-in from NIH Image J® software (Wayne Rasband, National Institutes of Health, USA). Data were expressed in cells/mm^2^.

Microglial cell proliferation was quantified on sections immunostained for the mitotic marker pH3 as well as BrdU in NL and PPT-lesioned animals at 2, 3, and 7dpl in the case of pH3 and at 7dpl in the case of BrdU. For pH3, a minimum of three WT and three GFAP-IL6Tg animals per group were analyzed, whereas in the case of BrdU, five WT and six GFAP-IL6Tg animals were used. The number of both pH3^+^ and BrdU^+^ cells in the ML of DG was manually counted on 20 (for pH3) and 10 different sections (for BrdU) per animal using a × 20 objective. Data were averaged and represented as pH3^+^ cells/section or BrdU^+^ cells/section.

To evaluate T lymphocyte infiltration, sections stained for CD3 were used. At least three WT and three GFAP-IL-6Tg NL animals and at 2, 3, 7, and 14dpl were used. All CD3^+^ cells in the ML of DG were manually counted on 20 sections per animal using a × 20 objective. Data were averaged and represented as CD3^+^ cells/section.

To analyze axonal sprouting, sections stained with the sulfide-silver staining were used. At least three WT and five GFAP-IL6Tg NL animals and at 14 and 21 dpl were analyzed. A total of 24 photographs from 8 different hippocampal sections per animal were captured at × 20 magnification using the same device and software referred above. The percentage of area occupied was obtained using the analySIS® software.

### Morphometric analysis

Morphometric analysis of microglial cells was done on sections immunolabeled for Iba1. At least three WT and three GFAP-IL6Tg animals were analyzed. For each animal, a total of 30 representative microglial cells were randomly chosen from 9 different photographs from the ML of the DG of the hippocampus and photographed at × 40 magnification. Using the analySIS® software, individual cells were isolated and different parameters including the area occupied, the sphericity (value equal to 1 indicates spherical shape and low values increased elongation), the shape factor (high values indicate round shape and low values ramified morphology), and the elongation (value equal to 1 indicates round morphology and high values increased elongation) were recorded for each cell.

### Flow cytometry analysis

The phenotype of the microglia/macrophage populations and the T cell infiltration in NL and lesioned animals (at 3 and 7 dpl) were analyzed using flow cytometry as previously described [1].

Briefly, animals were anaesthetized and intracardially perfused for 1 min with 0.1 M phosphate buffer solution (PBS), brains removed, and the entire ipsilateral hippocampus was quickly dissected out. In order to obtain a cell suspension, samples were dissociated through 140 μm and 70 μm meshes and digested for 30 min at 37 °C using collagenase type IV (17104-019, Life Technologies) and DNAsa I (D5025, Sigma). After that, each cellular suspension was centrifuged at RT for 20 min at 2400 rpm in a discontinuous density Percoll gradient (17-0891-02, Amersham-Pharmacia) between 1.03 and 1.08 g/mL. Myelin in the upper layer was removed. Cells in the interphase and in the clear upper-phase were collected, washed in PBS + 2% serum, and the Fc receptors were blocked by incubation for 10 min in a solution of purified CD16/32 diluted in PBS + 2% serum. Afterwards, cells were labeled for 30 min at 4 °C with the following four combinations of cell surface antibodies: (1) anti-CD11b-APC-Cy7, anti-CD45-PerCPCy5, anti-CD11c-PE, anti-MHCII-FITC, anti-CD86-PE-Cy7, anti-CD80-APC; (2) anti-CD11b-APC-Cy7, anti-CD45-PerCPCy5, anti-Ly6C-FITC, anti-F480-APC, and anti-ICOSL-PE; (3) anti-CD3-FITC, anti-CD4-APC-Cy7, and anti-CD8-PerCPC; and (4) anti-CD3-FITC, anti-CD4-APC-Cy7, anti-Tbet-PerCPCy5.5, anti-RORγt-APC, anti-Foxp3-PE-Cy7, and anti-Gata3-PE (Table 3). In parallel, isotype-matched control antibodies for the different fluorochromes (BD Pharmingen) were used as negative control and a cell suspension of splenocytes as positive control. Data were extrapolated as number of cells using the Cyto Count™ fluorescent beads following the manufacturer’s instructions (S2366, Dako Cytomation). Finally, cells were acquired using a FACS Canto flow cytometer (Becton Dickinson, San Jose, CA) and results analyzed using the FlowJo® software. The analysis was performed separately for each animal without any pooling.

**Table 3:** Antibodies used in flow cytometry

Target antigen		Format	Dilution	Cat Number	Manufacturer
Fc blocker	CD16/32	Purified	1:250	553142	BD Pharmingen
Primary antibodies	CD11b	APC-Cy7	1:400	557657	BD Pharmingen
	CD45	PerCPCy5	1:400	557235	BD Biosciences
	MHCII	FITC	1:400	553623	BD Pharmingen
	CD11c	PE	1:400	557401	BD Pharmingen
	Ly6C	FITC	1:400	553104	BD Pharmingen
	ICOSL	PE	1:400	12-5985-82	BD Bioscience
	CD86	PE-Cy7	1:400	560582	BD Pharmingen
	CD80	APC	1:400	560016	BD Pharmingen
	CD11c	PE	1:400	557401	BD Pharmingen
	F4/80	APC	1:400	17-4801-82	eBioscience
	CD3e	FITC	1:400	553062	BD Pharmingen
	CD4	APC-Cy7	1:400	552051	BD Pharmingen
	CD8	PerCP	1:400	553036	BD Pharmingen
	T-bet	PerCPCy5.5	1:400	45-5825	eBioscience
	RORγt	APC	1:400	17-6988-82	eBioscience
	Foxp3	PECy7	1:400	25-5773-80	eBioscience
	Gata3	PE	1:400	560074	BD Pharmingen

### Tissue processing for protein analysis

Animals used for protein analysis were i.p anaesthetized (as described above) and perfused for 1 min with cold 0.1 M PBS (pH 7.4). Subsequently, the entire ipsilateral hippocampus was dissected out, snap frozen individually in liquid nitrogen, and stored at − 80 °C. Total protein was extracted by solubilization of samples on lysis buffer, containing 25 mM HEPES, 2% Igepal, 5 mM MgCl_2_, 1.3 mM EDTA, 1 mM EGTA, 0.1 M PMSF, and protease (1:100, P8340, Sigma Aldrich) and phosphatase inhibitor cocktails (1:100, P0044, Sigma Aldrich), for 2 h at 4 °C. After solubilization, samples were centrifuged at 13,000 rpm for 5 min at 4 °C and the supernatants collected. The hippocampus from each animal was analyzed separately. Total protein concentration was determined with a commercial Pierce® BCA Protein Assay kit (#23225, Thermo Scientific) according to manufacturer’s protocol. Protein lysates were stored at − 80 °C until used for the protein microarray analysis.

### Cytokine analysis

The cytokines IL-2, IL-6, IFN-γ, IL-1β, IL12p70, IL-17, IL-10, IL-13, IL-9, IL-5, and IL-4 and the chemokines CXCL10 and CCL2 were analyzed using a Milliplex® MAP Mouse Cytokine/Chemokine kit (#MCYTOMAG-70K, Merck Millipore) according to the manufacturer’s instructions. Briefly, 25 μL of each hippocampus extract with a final total protein concentration of 2.5 μg/μL was added to the plate, along with the standards in separate wells, containing 25 μL of custom fluorescent beads and 25 μL of matrix solution and incubated overnight at 4 °C in a plate-shaker (750 rpm). After two washes with wash buffer (WB), the plate was incubated with 25 μL of detection antibodies for 30 min at RT followed by incubation with 25 μL of Streptavidin-Phycoerythrin for 30 min at RT in a plate-shaker (750 rpm). Finally, the plate was washed two times with WB and 150 μL of Drive fluid was added. Luminex® MAGPIX® device with the xPONENT® 4.2 software was used to read the plate. Data were analyzed using the Milliplex® Analyst 5.1 software and expressed as pg/mL of protein.

### Tissue processing for serum samples

Animals used for serum samples were i.p anaesthetized (as described above) and blood extraction were performed by cardiac puncture. After blood extraction, samples were centrifuged at 10,000 rpm for 5 min at 4 °C and the supernatants collected. Finally, samples were stored at − 80 °C until used for the ELISA analysis.

### IL-6 ELISA

The IL-6 levels in serum were analyzed using a Mouse IL-6 Uncoated ELISA kit (88-7064, Invitrogen) according to the manufacturer’s instructions. Briefly, the plate was coated overnight at 4 °C and after washes; wells were blocked for 1 h at RT. After several washes, 100 μL of each serum extraction and standards, in separated wells, were added and incubated overnight at 4 °C. After reagents incubations, and the addition of TMB solution and Stop solution, results were read at 450 nm in the microplate reader VarioskanTM Lux (ThermoFisher Scientific) and data were expressed as pg/mL of protein.

### Statistical analysis

Statistics were performed using the Graph Pad Prism 5.0 ® software. To study the differences between NL WT and NL GFAP-IL6Tg animals unpaired Student’s *T* test was used, while two-way ANOVA followed by Tukey’s post hoc analysis was used to study the effect of the lesion in both genotypes. All experimental values were expressed as mean values ± SD.

## Results

### Astrocyte-targeted IL-6 production modifies the number and morphology of microglia/macrophage cell populations

#### Analysis of microglia/macrophage cell distribution and morphology

To investigate possible changes in the brain cyto-architecture and cell distribution, by astrocyte-targeted IL-6 production in the CNS, a microscopic study was performed on toluidine blue sections (Suppl Fig. 2). In NL conditions, both WT and GFAP-IL6Tg animals showed the same distribution of cells through the ML. However, GFAP-IL6Tg showed an increased number of cells. After PPT, the presence of cells increased in the outer molecular layer (OML) and medial molecular layer (MML) in both WT and GFAP-IL6Tg animals from 3 to 7 dpl. In parallel to changes found in the OML and the MML, both WT and GFAP-IL6Tg animals showed a progressive reduction of cells in the inner molecular layer (IML) along the different time-points (from 3 to 14 dpl) (Suppl Fig. 2).

The possible changes in distribution and morphology of the microglia/macrophage cell population evoked by the transgenic production of IL-6 were analyzed using Iba1 IHC.

**Fig. 1:**
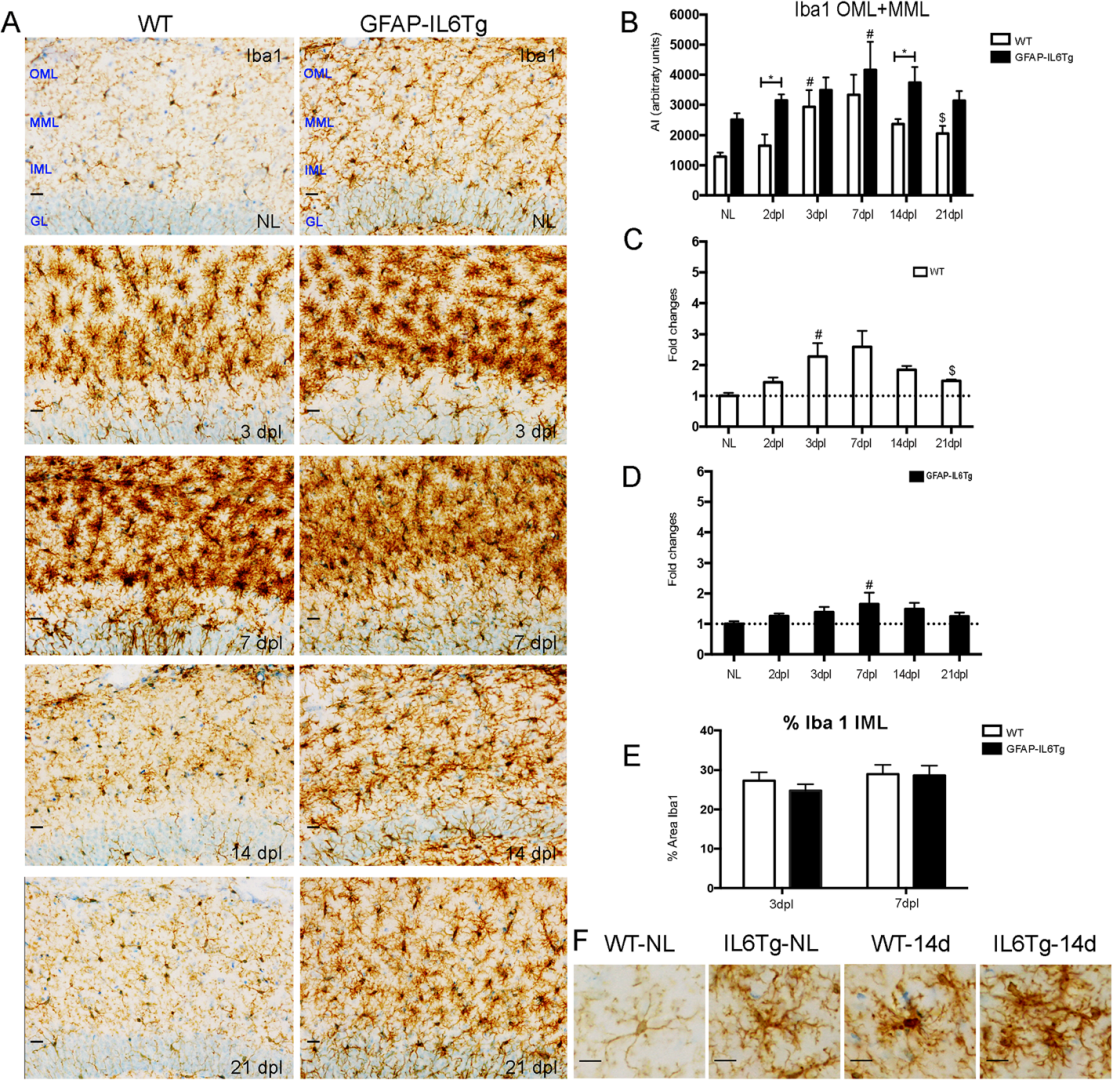
Microglial cell activation. Representative images from WT and GFAP-IL6Tg mice showing Iba1 staining in the granular (GL) as well as the inner, medial, and outer molecular layer (IML, MML, and OML, respectively) of the dentate gyrus (DG) in non-lesioned (NL) and PPT-lesioned hippocampus from 3 to 21dpl. Scale bar = 20 μm (**a**) and 10 μm (**f**). **b** Graph showing the time course of Iba1 immunostaining, expressed as AI (area x intensity). In comparison with WT, GFAP-IL6Tg animals showed increased levels of Iba1 at 2 and 14dpl. **c**, **d** Graphs showing the fold change increase of Iba1 expression in comparison with the corresponding NL animals in both WT and GFAP-IL6Tg mice. **e** Graph showing the area occupied by Iba1 in the IML in both WT and GFAP-IL6Tg at 3 and 7 dpl. A minimum of 3 animals per genotype and experimental time-point were analyzed. A total of 9 photographs from 3 different hippocampal sections were used. Data are represented as mean ± SD. The significances are represented as #*p* ≤ 0.05 vs. NL of respective group; $*p* ≤ 0.05 vs. 7dpl of respective group. Significant differences between genotypes are represented as *p ≤ 0.05

In NL WT animals, microglial cells showed the characteristic ramified morphology throughout the ML of the DG (Fig. 1a, f), whereas in the GFAP-IL6Tg mice, microglia had a significant increase of Iba1 immunoreactivity (Fig. 1b) as well as morphological changes mainly characterized by a greater number and thickness of ramifications and an increase in the number of processes (Fig. 1a). These qualitative morphological changes were quantified by a morphometric analysis of different parameters such as the area, shape factor, the elongation, and sphericity of individual Iba1^+^ microglial cells. Confirming the qualitative findings, GFAP-IL6Tg animals showed a significant increase in the area occupied by individual microglial cells (Suppl Fig. 3A). Moreover, microglial cells in transgenic animals showed a higher ramified morphology, as indicated by less values of shape factor (Suppl Fig. 3D), and a rounded morphology (Suppl Fig. 3B-C).

After PPT, Iba1 immunoreactivity in WT animals increased at 3 dpl and decreased at 21 dpl, whereas in GFAP-IL6Tg animals Iba1 showed a later increase at 7 dpl and remained stable until 21 dpl (Fig. 1a, b). Noticeably, at 2 and 14 dpl (Fig. 1b), GFAP-IL6Tg mice showed higher levels of microglial cell Iba1 compared to WT (1651 ± 376.6 vs. 3145 ± 202.3 AI, WT/2dpl vs. Tg/2dpl, *p* = 0.014 and 4160 ± 939.0 vs. 2374 ± 63.5 AI, WT/14dpl vs. Tg/14dpl, *p* = 0.032). To quantify the dynamics of microglial activation following PPT lesion in both WT and GFAP-IL6Tg animals, the fold-changes with respect to the corresponding NL animals were calculated (Fig. 1c, d). This revealed that, although the levels of Iba1 in GFAP-IL6Tg animals were higher than in WT, the upregulation of Iba1 with respect to its basal levels was less pronounced in the transgenic animals than in WT, at specific time-points along the progression of the PPT lesion. Thus, while WT animals showed around a 3-fold increase of Iba1 at 7dpl in comparison with their basal levels (Fig. 1c), in GFAP-IL6Tg mice, this increase remained less than 2-fold at any time-point analyzed (Fig. 1d). Finally, no differences in the area occupied by Iba1 were observed in the IML of both WT and GFAP-IL6Tg animals after PPT (Fig. 1e).

In addition to changes in Iba1 levels, after PPT, both WT and GFAP-IL6Tg mice showed alterations in microglial cell morphology with some differences noted. At early time-points (3 and 7dpl), microglial cells in both genotypes showed the typical “bushy” shapes characterized by short and stubby ramifications (Suppl Fig. 3). At later time-points (14 and 21 dpl), microglial cells in both WT and GFAP-IL6Tg animals returned to ramified morphologies, although comparatively, microglia in the transgenic animals displayed a greater number and thickness of ramifications than WT at these time-points (Fig. 1f).

#### Analysis of microglia/macrophage cell density

To evaluate possible changes in the number of microglia/macrophages IHC for the transcription factor Pu.1, a specific myeloid marker [40], was used.

In parallel to changes in microglia/macrophage distribution and morphology, our observations revealed around a 2-fold increase in the number of Pu.1^+^ cells in the ML of the DG in NL GFAP-IL6Tg animals in comparison with NL WT (Suppl. Fig. 4 and Suppl. Fig. 11B).

After PPT, the number of Pu.1^+^ cells in WT animals increased progressively from 2 to 3 dpl, whereas in GFAP-IL6Tg animals, this increase was observed only at 3 dpl (Suppl. Fig. 4A). At later time points, in WT animals, Pu.1^+^ cells exhibited a marked decrease at 14 dpl, whereas in GFAP-IL6Tg mice remained stable until 21 dpl (Suppl. Fig. 4A). Consequently, in transgenic animals, the number of Pu.1^+^ cells at 14 dpl was significantly higher than WT (759 ± 292.8 vs. 1384 ± 305.2 number of cells, WT/14dpl vs. Tg/14dpl, *p* = 0.010) (Suppl. Fig. 4B). Notably, when dynamics of Pu.1 upregulation were compared with the corresponding NL, it was found again that WT animals had a nearly 5-fold increase in the number of Pu.1^+^ cells in the ML of the DG (Suppl. Fig. 4C), whereas GFAP-IL6Tg animals showed only a 2-fold increase in Pu.1^+^ cells compared with NL GFAP-IL6Tg animals (Suppl. Fig. 4D).

### Astrocyte-targeted IL-6 production increases microglial cell proliferation after PPT but has no effects on cell death

**Fig. 2:**
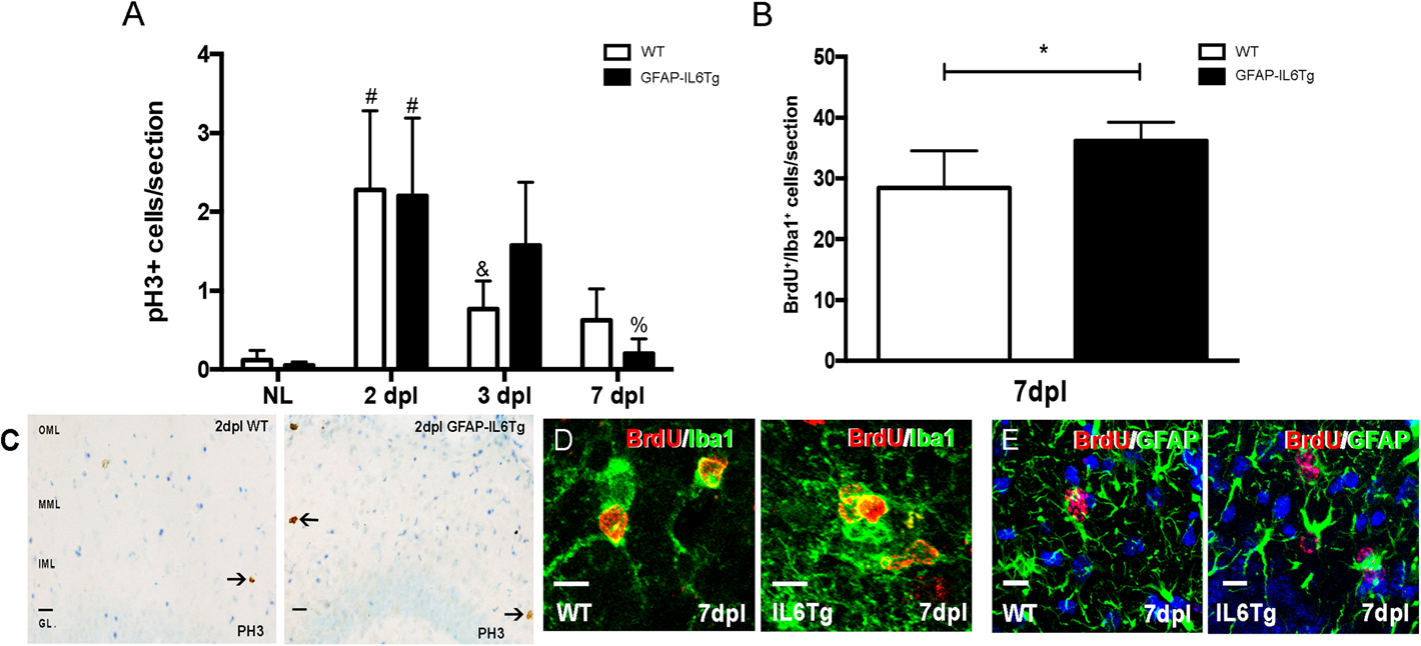
Microglial proliferation. **a** Graph showing the quantification of pH3^+^ cells in non-lesioned (NL) and PPT-lesioned animals from 2 to 7 dpl, in WT and GFAP-IL6Tg mice. **b** Graph showing the quantification of BrdU^+^/Iba1^+^ cells in PPT-lesioned animals at 7dpl in WT and GFAP-IL6Tg animals. Note that the total number of BrdU^+^ proliferating cells is significantly higher in transgenic animals. **c** Representative images of pH3^+^ cells in WT and Tg animals at 2 dpl. Scale bar = 20um. **d** Representative images of double IHC combining BrdU (red) and Iba1 (green). **e** Representative images of double IHC combining BrdU (red) and GFAP (green). Scale bar = 10 μm. For pH3, a minimum of three WT and three GFAP-IL6Tg animals per group were analyzed, whereas in the case of BrdU five WT and six GFAP-IL6Tg animals were used. Data are represented as mean ± SD. The significances are represented as #*p* ≤ 0.01 vs. NL of respective group; &*p* ≤ 0.05 vs. 2dpl of respective group and %*p* ≤ 0.01 vs. 3dpl of respective group. Significant differences between genotypes are represented as *p ≤ 0.05

In order to study whether changes observed in microglial cell numbers in GFAP-IL6Tg animals were related to changes in either proliferation and/or microglial cell death, pH3 and BrdU detection was used for the analysis of proliferation (Fig. 2) and activated caspase-3 and TUNEL for apoptosis evaluation.

In both NL WT and NL GFAP-IL6Tg animals, few pH3^+^ cells were observed in the ML of the DG, with no differences between genotypes. After PPT, although the peak of proliferation was at 2 dpl in both genotypes, in WT animals the number of pH3^+^decreased at 3 dpl, whereas in GFAP-IL6Tg animals the number of pH3^+^ cells decreased later on, at 7 dpl (Fig. 2a, c). Furthermore, to explore whether, additionally to the dynamics of proliferation, IL-6 overproduction produced modifications in the total amount of proliferating cells, a daily injection of BrdU was performed during 7 days. This study demonstrated that the total number of accumulated BrdU^+^/Iba1^+^ cells until 7dpl was significantly higher in GFAP-IL6Tg animals than in WT (23.06 ± 7.39 vs. 36.19 ± 6.16 number of cells, WT/7dpl vs. Tg/7dpl, *p* = 0.049) (Fig. 2b). Using double stain immunohistochemistry, it was determined that parenchymal BrdU^+^ cells in both WT and GFAP-IL6Tg animals corresponded mainly to Iba1^+^ microglia/macrophages, although some scattered proliferating astrocytes (BrdU^+^/GFAP^+^) were also found (Fig. 2d, e).

Regarding microglial cell death, no staining for either active caspase-3 or TUNEL was detected in the dennervated ML of the DG in either WT or GFAP-IL6Tg animals from 2 to 21 dpl (data not shown).

### Astrocyte-targeted IL-6 production increases the number of monocytes/macrophages

Flow cytometry was used to assess the number of microglia/macrophage populations induced by the transgenic production of IL-6 (Suppl. Fig. 5). The microglia/macrophage population was identified based on the positive CD11b expression in combination with differential expression levels of CD45 (Suppl. Fig. 5A). This helped in differentiating homeostatic from activated microglia as well as macrophages. Thus, ramified or homeostatic microglia were identified as CD11b^+^/CD45^low^ and activated microglia as CD11b^+^/CD45^int^. In this study, the term CD11b^+^/CD45^low/int^ has been used to refer to the population of homeostatic and activated microglia jointly. Moreover, the CD11b^+^/CD45^high^ population was identified, which may include highly activated microglia, monocytes, macrophages, and dendritic cells. The percentage of CD11b^+^/CD45^high^ population, in the GFAP-IL6Tg, was higher than in WT at all time points analyzed (Suppl. Fig. 7).

Taking in account the total number of cells, in NL animals, our results showed a significant increase of both CD11b^+^/CD45^low/int^ (318.3 ± 124.7 vs. 524.3 ± 154.5 number of cells, WT/NL vs. Tg/NL, *p* = 0.08) and CD11b^+^/CD45^high^ populations (7.50 ± 3.06 vs. 45.57 ± 14.06 number of cells, WT/NL vs. Tg/NL, *p* = 0.001) in GFAP-IL6Tg animals (Suppl. Fig. 11C and D).

After PPT, GFAP-IL6Tg animals exhibited an increase in the number of both CD11b^+^CD45^low/int^ and CD11b^+^/CD45^high^ cell population at 7dpl when compared to their own NL, while in WT animals no changes were observed. Interestingly, the levels of CD45 (mean fluorescence intensity) were always higher in the CD11b^+^/CD45^low/int^ cell population of GFAP-IL6Tg animals than in WT (Suppl. Fig. 5B). However, in CD11b^+^/CD45^high^ cell population, no differences between genotypes were observed (Suppl. Fig. 5B).

Using CD45 immunohistochemistry, it was confirmed that GFAP-IL6Tg animals showed a higher activated microglial phenotype (Suppl. Fig. 5C). Also, after PPT, scattered CD45^+^ cells with round morphology were observed through the ML of the FD in both experimental groups that could correspond to T cells and/or infiltrated monocytes (Suppl. Fig. 5C black arrows).

Taking into account that the population of CD11b^+^/CD45^high^ cells includes monocytes, macrophages, dendritic cells, and highly activated microglia, we next addressed whether the changes observed in the number of microglia/macrophages were due specifically to variations in the number of monocytes or rather to the presence of highly activated microglia. For this purpose, the expression of the macrophage marker F4/80 and the monocytic marker Ly6C were evaluated in the CD11b^+^/CD45^low/int^ and CD11b^+^/CD45^high^ cell populations (Suppl. Fig. 6).

In NL animals, the CD11b^+^/CD45^low/int^ cell population in GFAP-IL6Tg animals had a higher number of F4/80^+^ cells, but not Ly6C^+^ cells compared to WT (Suppl. Fig. 11E). On the other hand, the CD11b^+^/CD45^high^ cell population showed an increased number of cells expressing F4/80 (2.69 ± 2.36 vs. 56.44 ± 32.48 number of cells, WT/NL vs. Tg/NL, *p* = 0.046) and Ly6C (1.54 ± 0.69 vs. 5.07 ± 2.30 number of cells, WT/NL vs. Tg/NL, *p* = 0.026) in transgenic animals than WT (Suppl. Fig. 11F and G).

After PPT, in WT animals, no statistically significant differences in the number of F4/80^+^ and Ly6C^+^ cells were found after PPT in comparison with the corresponding NL controls. However, in GFAP-IL6Tg animals, the number of Ly6C^+^ cells increased a 3 dpl within the CD11b^+^/CD45^high^ cell population (Suppl. Fig. 6C). Comparisons between genotypes revealed no significant variations in the number of either F4/80^+^ or Ly6C^+^ cells within the CD11b^+^/CD45^low/int^ cell population (Suppl. Fig. 6A and C). However, a greater number of F4/80^+^ cells in the CD11b^+^/CD45^high^ cell population was found at 3 and 7dpl in GFAP-IL6Tg animals (Suppl. Fig. 6A).

Furthermore, analysis of the levels of F4/80, calculated using the mean fluorescence intensity, demonstrated a higher F4/80 in the CD11b^+^/CD45^high^ cell population of GFAP-IL6Tg animals in both NL and at 7 dpl (Suppl. Fig. 6B). However, in the CD11b^+^/CD45^high^ cell population, the levels of Ly6C in NL transgenic animals were less than WT (4273 ± 148.1 vs. 2144 ± 523.9 mean fluorescence, WT/NL vs. Tg/NL, *p* = 0.0001) (Suppl. Fig. 6D).

### Astrocyte-targeted IL-6 production modifies the phenotype of CD11b^+^/CD45^high^ cell population

**Fig. 3:**
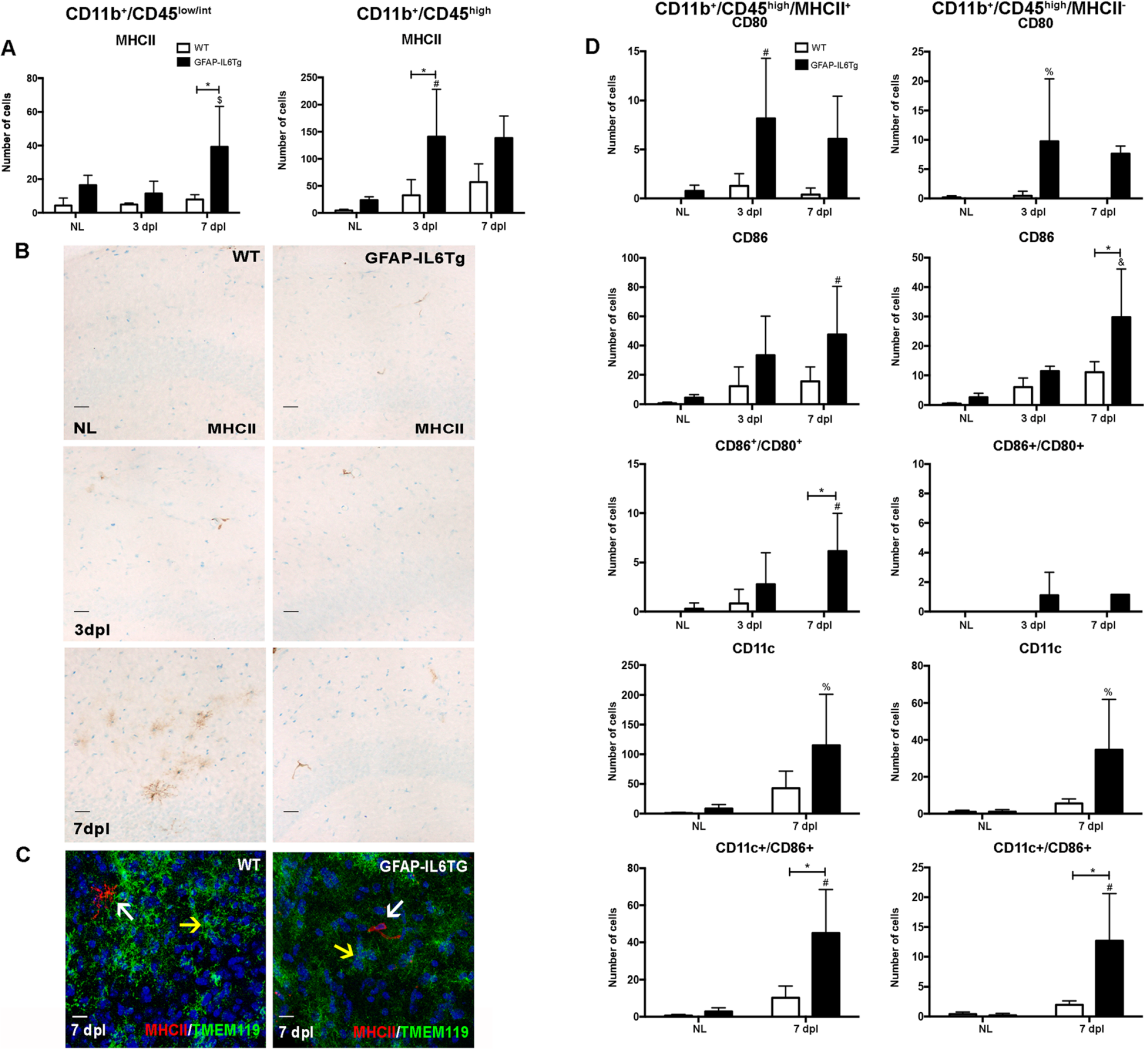
Phenotypic analysis of microglia and macrophage populations. **a** Graphs showing the number of MHCII^+^ cells in the CD11b^+^/CD45^low/int^ and CD11b^+^/CD45^high^ cell population. **b** Representative images from WT and GFAP-IL6Tg mice showing MHCII staining in the ML of the DG in NL and PPT-lesioned hippocampus from 3 to 7 dpl. Scale bar = 20 μm. **c** Representative images, from WT and GFAP-IL6Tg mice, of double IHC combining MHCII (red) and Tmem119 (green) at 7 dpl. White arrows indicate MHCII^+^/Tmem119^−^ cells and yellow arrow indicate microglia Tmem119^+^ Scale bar = 5 μm. **d** Graphs showing the number of CD80^+^, CD86^+^, and CD11c^+^ cells in the CD11b^+^/CD45^high^/MHCII^+^ and CD11b^+^/CD45^high^/MHCII^−^ cell populations. A minimum of three WT and three GFAP-IL6Tg animals per group were used for this study. Data are represented as mean ± SD. The significances are represented as #*p* ≤ 0.05 vs NL of respective group; %*p* ≤ 0.1 vs. NL of respective group; &*p* ≤ 0.001 vs. NL of respective group and $*p* ≤ 0.05 vs. 3dpl of respective group. Significant differences between genotypes are represented as *p ≤ 0.05

In order to investigate the putative changes that transgenic IL-6 production may exert on the phenotype of the microglia and macrophage cell populations, the expression of different cell activation markers mostly related to antigen presentation, including MHCII, CD80, CD86, ICOSL, and CD11c, in both CD11b^+^/CD45^low/int^ and CD11b^+^/CD45^high^ populations was studied by flow cytometry (Fig. 3 and Suppl. Figs. 9 and 12).

In NL animals, in both WT and GFAP-IL6Tg animals, a small population of CD11b^+^/CD45^low/int^ cells expressed MHCII (WT: 1.2 ± 0.6%; Tg: 3.3 ± 0.7%) and ICOSL (WT: 0.22 ± 0.1%; Tg: 0.1 ± 0.08%) (Fig. 3a and Suppl. Fig. 9A), whereas the number increased in the CD11b^+^/CD45^high^ population (WT: 53.5 ± 9.4%; Tg: 53.23 ± 6%) (WT: 34.8 ± 5.4%; Tg: 22.4 ± 0.7%) (Fig. 3a and Suppl. Fig. 9B).

The number of cells expressing MHCII^+^ in both populations was always significantly higher in NL GFAP-IL6Tg animals than in NL WT (Fig. 3a and Suppl. Fig. 11H-I). In the case of the ICOSL population, NL GFAP-IL6Tg animals only showed a significant increase in the CD11b^+^/CD45^high^ population compared with WT (Suppl. Fig. 11J).

After PPT, an increase in the number of both CD11b^+^/CD45^low/int^/MHCII^+^ and CD11b^+^/CD45^high^/MHCII^+^ population was only observed in GFAP-IL6Tg animals (Fig. 3a). Moreover, in GFAP-IL6Tg animals, the number of ICOSL^+^ cells increased at 7 dpl within the CD11b^+^/CD45^low/int^ and CD11b^+^/CD45^high^ population, whereas in WT no changes were observed (Suppl. Fig. 9A-B).

Using MHCII immunostaining, MHCII^+^ cells in the ML of the DG were morphologically identified as perivascular cells in both WT and transgenic animals in NL and after PPT (Fig. 3b). These cells had an elongated morphology and stained with CD206 a marker commonly used to identify perivascular macrophages (Suppl. Fig. 8A). Furthermore, using double immunofluorescence with laminin, the location of these MHCII^+^ cells in the perivascular space was established (Suppl. Fig. 8B). Noticeably, at 7 dpl, we observed a subpopulation of MHCII^+^/Tmem119^−^ ramified microglia in the parenchyma of WT animals but not in GFAP-IL6Tg mice (Fig. 3c).

Taking into account that the major differences observed in terms of MHCII was in the CD11b/CD45^high^ population, we focused on the study of the co-expression of CD80, CD86, and CD11c with MHCII only in this population (Fig. 3d). In NL conditions, in contrast to WT animals in which these populations were not detected, a small population of CD80^+^ cells (3 ± 1.1%) and a higher number of CD86^+^ cells (16.3 ± 4.8%) was observed within the CD11b^+^/CD45^high^/MHCII^+^ population in GFAP-IL6Tg animals. In addition, a population of CD11b^+^/CD45^high^/MHCII^−^/CD86^+^ was found in NL transgenic animals (Fig. 3d).

In GFAP-IL6Tg animals, it was observed an increase of cells expressing CD80 or CD86 (Fig. 3d), either with or without MHCII. However, the number of cells co-expressing CD80 and CD86 only increased within the CD11b^+^/CD45^high^/MHCII^+^ population. Furthermore, populations of MHCII^+^/CD11c^+^ and MHCII^-^/CD11c^+^ cells, with or without CD86, were observed at 7 dpl in WT (Fig. 3d). Moreover, the number of CD11c^+^/CD86^+^ cells either with or without MHCII was always higher in GFAP-IL6Tg mice than in WT (Fig. 3d).

As mentioned above, no CD80^+^, CD86^+^, and cells co-expressing both markers were observed in the CD11b^+^/CD45^low/int^/MHCII^+^ population at any time-point analyzed in any genotype (data not shown).

### Astrocyte-targeted IL-6 production promotes T cell infiltration after PPT

Another aspect was whether transgenic production of IL-6 was able to modify the infiltration and/or differentiation of T-cells after PPT.

**Fig. 4:**
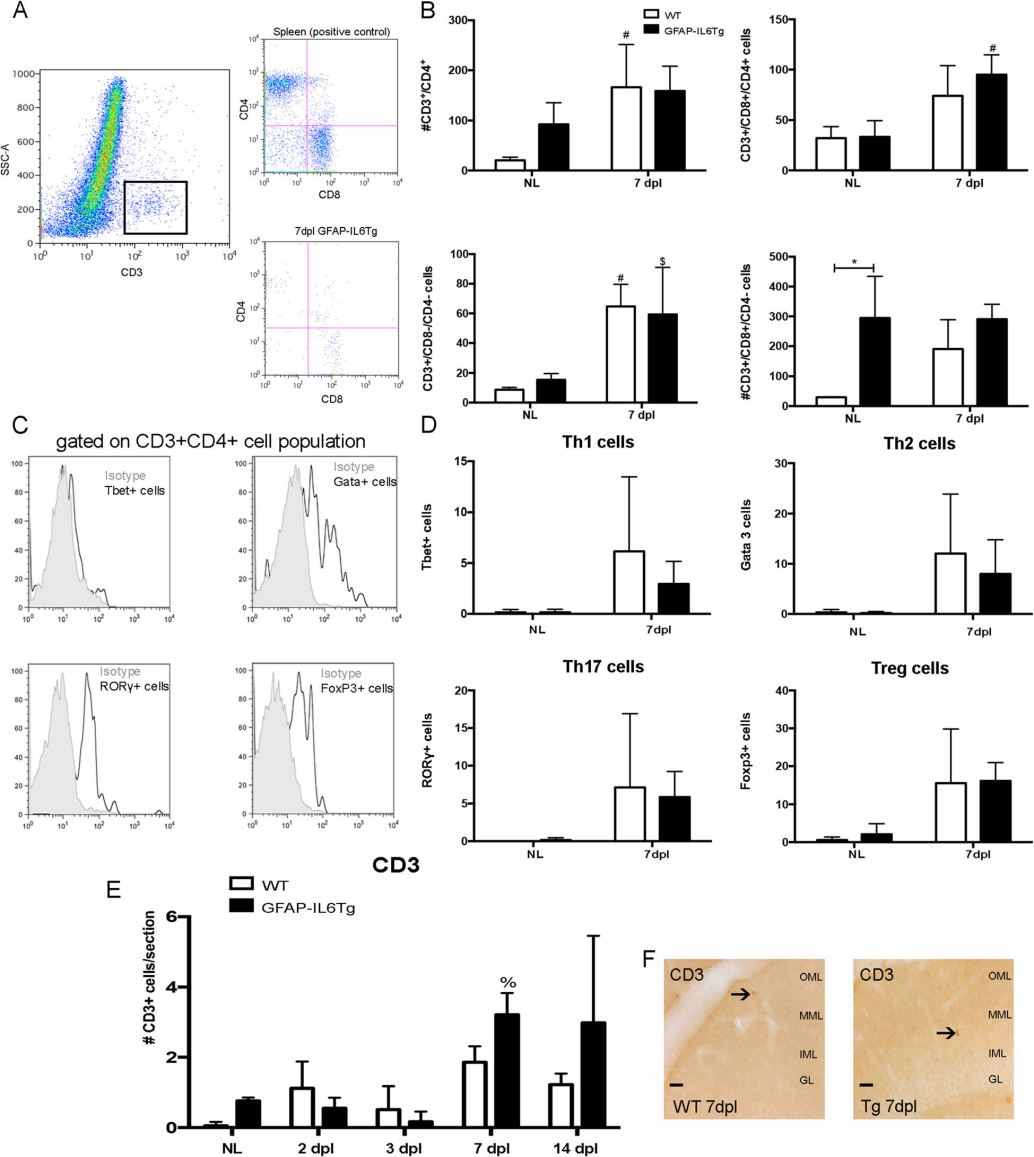
T cell infiltration after PPT. **a** Representative dot plot of SSC/CD3 expression of cells obtained from the lesioned WT animals. The square delimits the CD3^+^ cell population used in this study. Representative dot plot of CD3/CD4/CD8 cells obtained from the spleen, positive control (top), and hippocampus (bottom) of lesioned WT. **b** Graphs showing the number of CD4^+^, CD3^+^/CD4^+^/CD8^+^ cells, CD8^+^ and CD3^+^/CD4^-^/CD8^−^ in NL and at 7dpl. **c** Representative histogram-plot showing the expression of Tbet, Gata3, RORgt, and Foxp3 in the population of CD3^+^Th-cells in comparison to the corresponding isotype control, in which the positive staining was defined. **d** Graphs showing the number of the different subtypes of CD4^+^ T-helper lymphocytes: Tbet^+^, Gata 3^+^, RORg^+^, and Foxp3^+^ in NL and at 7dpl. **e** Graph showing the quantification of CD3^+^ immunostaining cells in non-lesioned (NL) and PPT-lesioned hippocampus from 2 to 14 dpl, in WT and GFAP-IL6Tg mice. **f** Representative images showing CD3 immunostaining in WT and GFAP-IL6Tg mice at 7 dpl. Scale bar = 20 μm. A minimum of four WT and four GFAP-IL6Tg animals per group were used for this study. Data are represented as mean ± SD. The significances are represented as #*p* ≤ 0.05 vs. NL of respective group; $*p* ≤ 0.1 vs. NL of respective group and %*p* ≤ 0.05 vs. 3dpl of respective group. Significant differences between genotypes are represented as *p ≤ 0.05

In order to study the dynamics of the lymphocyte populations, the number of CD4^+^ and CD8^+^ cells within the gated CD3^+^ T cell population was analyzed in the entire ipsilateral hippocampus by flow cytometry, in NL and in PPT-lesioned WT and GFAP-IL6Tg mice at 7dpl (Fig. 4a and b).

In comparison with NL WT, NL GFAP-IL6Tg animals showed a higher number of CD4^+^ T cells (20.44 ± 6.25 vs. 92.27 ± 43.29 number of cells, WT/NL vs. Tg/NL, *p* = 0.046), CD8^+^ T cells (29.62 ± 1.63 vs. 294.9 ± 139.9 number of cells, WT/NL vs. Tg/NL, *p* = 0.031), and CD3^+^/CD4^−^/CD8^−^ cells (8.73 ± 1.59 vs. 15.34 ± 4.27 number of cells, WT/NL vs. Tg/NL, *p* = 0.07) but similar numbers of CD3^+^/CD4^+^/CD8^+^ cells (Fig. 4b and Suppl. Fig. 11K and L).

After PPT, at 7 dpl, both experimental groups showed an increase in CD3^+^/CD4^−^/CD8^−^ T cells, whereas in the CD3^+^/CD4^+^/CD8^+^ population, only GFAP-IL6Tg animals showed an increase, and in the CD3^+^/CD4^+^ population was only observed in WT animals (Fig. 4b). No significant difference was found in any T cell population between WT and GFAP-IL6Tg mice (Fig. 4b).

Finally, the different subtypes of CD4^+^ T-helper lymphocytes were also determined by flow cytometry using antibodies against lineage-specific transcription factors (Fig. 4d). After gating in the CD3^+^CD4^+^ T-helper cell population, the number of Tbet^+^ (for Th1), Gata 3^+^ (for Th2), RORγ^+^ (for Th17) and Foxp3^+^ (for T-regulatory) cells was analyzed (Fig. 4c). In both NL conditions and after PPT, no significant differences in the number of Th-cell infiltration between the two genotypes were found (Fig. 4d).

The possible changes in the distribution and the pattern of recruitment of T cell population, in the ML of the DG, were analyzed using sections immunostained for CD3 (a pan T cell marker) of NL and in PPT-lesioned WT and GFAP-IL6Tg animals (Fig. 4f). In NL conditions, a higher number of CD3^+^ cells was detected in GFAP-IL6Tg animals (Suppl. Fig. 11M). After PPT, WT animals showed two waves of T cell infiltration, the first at 2 dpl and the second at 7 dpl. In GFAP-IL6Tg mice, we only detected an increase in lymphocytes at 7 dpl. Moreover, in both genotypes, the CD3^+^ cells were observed randomly distributed through the ML of the DG and the hilus (Fig. 4f). From 7 to 14dpl, the numbers of these cells did not change in either WT or GFAP-IL6Tg mice (Fig. 4e).

### Absence of T cell co-stimulatory molecules after PPT

In order to investigate the putative changes that transgenic IL-6 production may exert on the communication between T cells and microglia/macrophages, we analyzed CTLA-4 and CD28, two identified ligands for CD80 and CD86, in PPT-lesioned WT and transgenic mice at 7dpl. Our results showed no detectable CTLA-4 and CD28 in either WT or GFAP-IL6Tg mice at any time-point studied (data not shown).

### Astrocyte-targeted IL-6 production induces alterations in the cytokine/chemokine microenvironment after PPT

We also addressed whether the changes observed in transgenic animals correlated with variations in various cytokines involved in microglial cell activation and T cell differentiation, including pro-inflammatory cytokines such as IL-2, IL-6, IFN-γ, IL-1β, IL-12p70, and IL-17; anti-inflammatory cytokines such as IL-10, IL-13, IL-9, IL-5, and IL-4; and chemokines, related with leukocyte recruitment, like CXCL10 and CCL2 (Suppl. Fig. 10).

In NL conditions, when only animals of the two genotypes are compared, significant differences were observed in the presence of the pro-inflammatory cytokines IL-6 and IL-17 and the anti-inflammatory cytokines IL-10 and IL-13 in the nervous parenchyma (Suppl. Fig. 11N-Q). In contrast, no modifications of IL-6 levels in the serum were found in transgenic animals (Suppl. Fig. 10N).

After PPT, WT animals only showed induction of the CXCL10 and CCL2 chemokines, while no induction was detected in any of the cytokines studied at the different time-points analyzed (Suppl. Fig. 10L and M).

PPT-lesioned GFAP-IL6Tg animals showed, in relation to the WT, differences in the levels of pro-inflammatory and anti-inflammatory cytokines as well as in chemokines. In concordance with the transgene expression, the cytokine with the greatest difference was IL-6, which was at significantly higher levels at 3 and 7 dpl. In the case of pro-inflammatory cytokines, transgenic animals had significantly higher IL-1β at 3dpl (Suppl. Fig. 10D), but lower IFN-γ and IL-12p70 at 14dpl (Suppl. Fig. 10C and E). In the case of anti-inflammatory cytokines, GFAP-IL6Tg animals showed higher levels of IL-10 than WT at 7 dpl but had a significant decrease at 14dpl (Suppl. Fig. 10G). Finally, the GFAP-IL6Tg mice had a significant increase in the chemokines CXCL10 and CCL2 at 3dpl that decreased significantly at 7 and 14dpl. Levels of these chemokines were higher in transgenic animals than WT at 3dpl (Suppl. Fig. 10L and M).

### Astrocyte-targeted IL-6 production reduces collateral sprouting after PPT

**Fig. 5:**
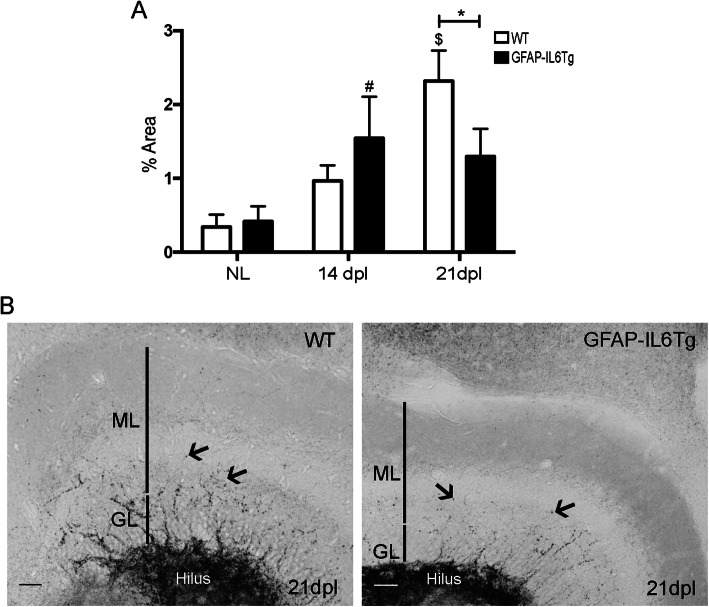
Collateral sprouting. **a** Graph showing the time course of the selenium-silver staining for zinc (arrows) expressed as proportion of occupied area. Note that GFAP-IL6Tg animals showed a reduction of Zinc^+^ staining at 21dpl. **b** Representative images from WT and GFAP-IL6Tg mice, at 21 dpl, showing staining in the ML of the DG (GL indicates granular layer). Scale bar = 20 μm. At least three WT and five GFAP-IL6Tg animals (NL animals and at 14 and 21dpl) were analyzed. A total of 24 photographs from 8 different hippocampal sections per animal were quantified. Data are represented as mean ± SD. The significances are represented as: #*p* ≤ 0.05 vs. NL of respective group and $*p* ≤ 0.01 vs. NL of respective group. Significant differences between genotypes are represented as: *p ≤ 0.05

Finally, it was explored whether the modifications detected in the population of microglia/macrophages, as well as in the infiltration of immune cells in GFAP-IL6Tg animals, affects the collateral sprouting after perforant path sectioning. Sprouting evaluation was done at later time points (14 and 21dpl) using the selenium-silver staining technique, which reveals glutamatergic zinc-rich buttons as in the hippocampal mossy fibers (Fig. 5) and other telencephalic pathways.

In NL animals, WT and GFAP-IL6Tg, large labeled buttons were densely packed in the hilus of the dentate gyrus and only few crossed the granular layer and reached the inner third of the molecular layer. After PPT, at 21 dpl, WT animals showed an increase in the area occupied by large, labeled buttons in the ML, indicative of sprouting, whereas in GFAP-IL6Tg animals, the increase was detected at 14 dpl (Fig. 5a). Consequently, at 21 dpl, transgenic animals showed significantly less labeled buttons in the ML compared with WT animals (2.32 ± 0.41 vs. 1.29 ± 0.36% area, WT/21dpl vs. Tg/21dpl, *p* = 0.049) (Fig. 5b and c).

## Discussion

The present study demonstrates that astrocyte-targeted IL-6 production causes significant alterations in the phenotype and density of the microglia/macrophage populations in the hippocampus of NL and PPT-lesioned animals. Moreover, an increase in the infiltration of CD11b^+^/CD45^high^/F4/80^+^ and CD11b^+^/CD45^high^/Ly6C^+^ monocytes and T lymphocytes were found in GFAP-IL6Tg mice. All these changes are linked to significant modifications in the cytokine/chemokine microenvironment and had a deleterious effect on axonal sprouting.

### Astrocyte-targeted production of IL-6 induces alterations in the microglia/macrophage, lymphocyte, and cytokine/chemokine microenvironment

The first interesting result of this study is that the chronic production of IL-6 increased the number of microglia/macrophages and modified their phenotype in homeostasis. Thus, 2.5 times more CD11b^+^/CD45^low/int^ and CD11b^+^/CD45^high^ cells were found. Although this increased number might reflect a higher infiltration of monocytes, the number of CD11b^+^/CD45^high^/F4/80^+^ and CD11b^+^/CD45^high^/Ly6C^+^ monocytes detected was not enough to explain the increase of microglia/macrophages in transgenic animals. In this regard, unpublished observations from our laboratory, demonstrated a higher number of microglia/macrophages during brain development in GFAP-IL6Tg mice, raising the possibility that the differences found in the number of cells in the adult would be linked to modifications from the post-natal period.

Regarding the phenotype of the microglia/macrophages, our study revealed that IL-6 promotes the activation of the microglial/macrophage cell population towards a more reactive macrophage-like cell, with higher expression of CD45 and F4/80 and a lower Ly6C. This low level of Ly6C could be interpreted as a stabilization of the entrance of these monocytes, whose levels decreased once in the parenchyma [38]. In concordance, IL-6 has been described as a potent activator of microglia/macrophage cells both in vitro and in vivo [15, 17, 21, 57, 91]. Also, astrocyte-targeted production of IL-6 induces an increase in the number of CD11b^+^/CD45^high^/MHCII^+^ cells and in cells expressing co-stimulatory molecules, including ICOSL, CD80 and CD86, suggesting a putative transformation into competent antigen presenting cells. Importantly, MHCII^+^ cells corresponded exclusively to perivascular macrophages and not parenchymal microglia, highlighting a necessity to study in more detail the role of these macrophages under homeostatic situations.

In agreement with previous studies describing increased basal lymphocyte infiltration in specific areas of the CNS of GFAP-IL6Tg mice [17, 18], we observed an increase in the number of T cells infiltrating the molecular layer of the DG in transgenic animals. The increase was due to the presence of CD4^+^ T-helper, CD8^+^ T-cytotoxic, and CD3^+^CD4^−^CD8^−^ γδ T cells. Studies in vitro have demonstrated the ability of IL-6 to induce a unique non-classical effector CD8^+^T cell subpopulation, called Tc17, characterized by a high production of IL-17 [69], and whose primary function is to contribute to inflammation and the recruitment of lymphocytes and myeloid cells [39]. In concordance, we found elevated IL-17 in NL GFAP-IL6Tg animals.

IL-6-induced modifications in the microglia/macrophage population and T cell infiltration were linked to a specific cytokine/chemokine profile, characterized by higher IL-6, IL-17, IL-13, and IL-10 production. As expected, major differences between WT and GFAP-IL6Tg animals were found for IL-6, which showed around a 5-fold increase in the GFAP-IL6Tg animals. As already commented, the upregulation of the pro-inflammatory cytokine IL-17 could be related with a higher number of CD8^+^ T cells and γδ T cells, one of the two cell-types that could produce this cytokine in the CNS [69, 93] and could be linked to alterations in the blood-brain barrier (BBB) [50], a well-described feature of these transgenic mice [14, 19] and the higher activation phenotype of microglia observed in basal conditions. Moreover, an effect of IL-17 on the upregulation of microglial activation has been reported, especially in demyelinating and autoimmune diseases [53, 96] coinciding with the higher activation profile observed in our transgenic animals in basal conditions. On the other hand, the higher levels of the classical anti-inflammatory cytokines IL-10 and IL-13 may be related to the increased number of perivascular macrophages and/or CD8^+^ T cells, two cell-type with the capacity to produce IL-10 [60, 63].

### Increased microglia/macrophage in GFAP-IL6Tg mice after PPT might be due to both proliferation and recruitment of bone-marrow derived monocytes

In agreement with previous studies in mice [29, 31, 43, 58, 66, 94], we found a progressive increase in the number of microglia/macrophages as well as an increase in Iba1 expression along with the progression of PPT in the molecular layer of the DG in both genotypes. However, in our study, the density of microglia/macrophages was higher in transgenic animals than in WT. This difference could be related to three changes after PPT: microglial proliferation; infiltration of blood-derived monocytes [5, 7, 59, 95], which transformed into microglial/macrophage cells [52]; and of microglial cells from adjacent non-deafferented areas, such as the CA1, the IML, and the hilus [29, 52, 59, 95].

Our results clearly showed an increase in the number of proliferating pH3^+^ cells, as well as in the total number of mitotic microglial cells (BrdU^+^), in GFAP-IL6Tg animals. This result was associated with an increase of IL-6, a potent inducer of microglial proliferation [56, 62, 80, 87]. Beyond proliferation, the recruitment of blood-derived monocytes paralleled this increase of microglia/macrophages in the transgenic mice. Thus, a higher number of CD11b^+^/CD45^high^ cells containing a high number of F4/80^+^ cells and Ly6C^+^ cells, widely used markers for monocyte/macrophages [49], was observed. As we will comment later, this greater presence of monocytes/macrophages correlated with the increased CCL2 production observed in GFAP-IL6Tg mice, a key chemokine involved in the leukocyte migration in the PPT-model [5], suggesting that not only proliferation but also infiltration is involved in the microglia/macrophages increase.

Another question is why the increased number of microglia/macrophages in PPT-lesioned transgenic animals remained at later time-points. One possibility is that the effects of chronic IL-6 production were related to the prevention of microglial/macrophage cell number restoration, by affecting either microglial cell death or monocyte exit from the CNS or even both. However, in concordance with previous studies [8, 78], we could not find any conclusive evidence of increased microglia/macrophage death, as we were not able to detect any evidence of apoptosis, monitored by active caspase-3 and TUNEL, at any time-point analyzed in any genotype.

### After PPT, astrocyte-targeted production of IL-6 promoted a shift in the phenotype of CD11b^+^/CD45^high^ cells and avoided the transformation of any parenchymal microglia into putative MHCII^+^ antigen presenting cells

Phenotypically, we demonstrated that, after PPT, part of the activated microglia/macrophage population in WT animals, expressed CD11c, MHCII, CD86, and ICOSL, molecules commonly associated with an antigen-presenting cell phenotype. These results are in agreement with already published papers reporting the expression of MHCII and CD86, but a lack of CD80 in microglia/macrophages after PPT [9]. The phenotype observed in lesioned GFAP-IL6Tg animals was similar to the reported in lesioned WT animals, and only a slight increase in the number of MHCII^+^/CD80^+^ and MHCII^+^/CD86^+^ cells at 7dpl and in the number of and MHCII^−^/CD86^+^ cells at 3 dpl were found in the CD11b^+^/CD45^high^ cell population of transgenic mice. Nevertheless, when we investigated the cells responsible for the expression of these molecules using immunohistochemistry, in the molecular layer of the DG, we found major differences between WT and GFAP-IL6Tg mice. In WT animals, in addition to perivascular macrophages, activated ramified microglia in the parenchyma expressed MHCII and CD11c and thus were equipped to engage an adaptive immune response. However, in GFAP-IL6Tg animals, microglia did not express MHCII or CD11c; this property is restricted to perivascular macrophages, suggesting that IL6-induced microglia are inadequate to act as antigen presenting cells and thus to drive an adaptive immune response, despite the higher presence of MHCII^+^ and CD11c^+^ cells. This reduction of MHCII observed in microglia might be explained by the higher expression of IL-10 observed at this same time-point in GFAP-IL6Tg mice and the ability of this cytokine to reduce MHCII on macrophages [68, 78].

In the present study, in both genotypes, all MHCII^+^ cells were TMEM119^−^, described as a specific marker of microglia that discriminates from blood-derived macrophages in the human brain [81]. However, several studies revealed that in homeostatic conditions, microglia express high levels of TMEM119, while in neurodegeneration disorders microglial cells show a great reduction [82]. In contrast, myeloid cells recruited into the lesions in the course of brain inflammation in rodents and humans do not express TMEM119. However, a recent study has been described that the regulation of microglial TMEM119 in MS is dependent on their inflammatory environment [92]. In this regard, the lack of expression of TMEM119 in MHCII^+^ cells does not provide evidence if these cells were resident microglia or infiltrated macrophages. Similarly, expression of P2Y12R, another marker commonly used to discriminate between macrophages and microglia, by its stable expression in microglia, was downregulated in our PPT-model [65] making not possible to use it for this purpose.

Another interesting result was the observation of a population of cells within the CD11b^+^/CD45^high^ population expressing CD86 and CD80 without MHCII in both WT and GFAP-IL6Tg mice. Noticeably, these populations were observed in transgenic animals already in homeostatic situations. This cellular phenotype has been associated with peripheral innate immune responses [74], and makes us hypothesize that transgenic animals presented a “primed microglia” with a desensitized profile in NL conditions, as suggested by other authors [72], and retained microglial activation in an innate immune phenotype after lesion.

### Astrocyte-targeted production of IL-6 increased lymphocyte recruitment after PPT without modifying their differentiation

Two different waves of T cell infiltration have been described in the PPT paradigm, the first wave at early time-points (2–3 dpl) and the second, later on, at 7 dpl [5, 6]. Our results showed that, in contrast to WT, GFAP-IL6Tg mice presented an increase in T cell infiltration in the molecular layer only at 7dpl, coinciding with the role attributed to IL-6 as a T cell recruitment molecule in peritoneal inflammation and in CNS-lesion models [3, 67] and a massive decrease in the recruitment of T cells after facial nerve injury in IL-6-deficient mice [36]. In most of these studies, the effects of IL-6 on lymphocyte recruitment have been related to alterations in the production of adhesion molecules and/or chemokines, including CXCL10 and CCL4, among others [67]. In the present study, we observed that after PPT GFAP-IL6Tg animals had elevated levels of CXCL10 and CCL2, two crucial chemoattractant proteins for both lymphocytes and monocytes in this paradigm [5], indicating that IL-6 may be promoting the infiltration of T cells by modifying the chemokine microenvironment.

Taking into consideration the role of IL-6 to drive lymphocyte differentiation, we analyzed the phenotype of infiltrated T cells in the PPT-lesioned hippocampus in more detail, quantifying the number of both CD8^+^ T-cytotoxic and CD4^+^ T-helper lymphocytes, as well as the different subtypes of T-helper cells. Until now, the specific subtypes of T lymphocytes infiltrating the parenchyma of the deafferented hippocampus in the PPT-paradigm were not characterized and to our knowledge, our study is the first to demonstrate the presence of a small population of the four subtypes of T-helper lymphocytes, Th1 (Tbet^+^), Th2 (GATA3^+^), Th17 (RORg^+^), and Treg (Foxp3^+^), in the PPT-lesioned hippocampus. No differences between WT and GFAP-IL6Tg animals were found in any T-helper populations analyzed or its expression of co-stimulatory molecules, suggesting that the increased number of lymphocytes found in GFAP-IL6Tg animals might be caused by the increased expression of CXCL10 and CCL2 rather than by specific action of IL-6 on T cell differentiation in this paradigm.

### Astrocyte-targeted production of IL-6 promoted alterations in the cytokine/chemokine profile associated with PPT

Astrocyte-targeted production of IL-6 markedly altered the cytokine/chemokine profile not only in the unlesioned state, as already discussed (see the first section of this discussion) but also after PPT-lesion. As expected, major differences between WT and GFAP-IL6Tg animals were found for IL-6, which showed around a 5-fold increase in the GFAP-IL6Tg animals in both unlesioned hippocampus and at early time-points after PPT. In addition to modifications in chemokines involved in the recruitment of macrophages and T cells, such as CXCL10 and CCL2, transgenic mice showed disturbances in the cytokine profile that may drive the type of the immune response. In this sense, astrocyte-targeted IL-6 production resulted in increased levels at early time-points (3 dpl) of IL-1β, cytokine more related with innate immune responses [28, 47, 63, 70, 88, 93]. Remarkably, cytokines like IL-17 and IL-13, whose levels we would expect to see increased given their upregulation in basal conditions, remained unaltered in lesioned animals. This fact suggests they are not crucial to the evolution of the PPT lesion and could explain the lack of differences in the shift of T lymphocyte subtypes observed between WT and GFAP-IL6Tg mice.

### Astrocyte-targeted production of IL-6 reduced axonal sprouting

Finally, we analyzed whether the modification of the immune response observed in GFAP-IL6Tg mice had any effect on the axonal sprouting of the remaining unlesioned axons of mossy cells, which typically occurs in the deafferented areas after PPT and is used as a marker of lesion outcome at later time-points [4, 25, 27, 33]. Our results indicated that GFAP-IL6Tg animals had lower axonal sprouting at 21 dpl than WT. Although in vitro IL-6 has a beneficial effect on axonal sprouting [44], in vivo our results showed a deleterious effect.

One putative explanation for the decreased axonal sprouting observed in transgenic mice may lie in the distinct cytokine/chemokine microenvironment observed. As we already commented, the molecules observed in WT animals are more related to the acquired immune response [9], whereas in GFAP-IL6Tg, the environment generated was more associated with a pro-inflammatory profile characteristic of the innate immune response.

Also, it is possible that some of the altered cytokines, and especially IL-6, act on the cells directly responsible for this axonal sprouting phenomenon. Astrocytes are the brain cells linked to axonal sprouting, as they secrete proteoglycans, such as neurocan, tenascin-C, brevican, widely demonstrated with an inhibitory role of axonal sprouting after PPT [26, 42, 85, 90]. Moreover, pro-inflammatory environments especially containing cytokines like TNF-α and IL-1β have been linked to the upregulation of these inhibitory proteoglycans, and thus with the inhibition of axonal outgrowth [41, 85]. More studies analyzing the composition of the extracellular matrix associated with PPT in GFAP-IL6Tg animals are needed to confirm this hypothesis.

Taking into consideration all the results observed in NL GFAP-IL6Tg mice, together with the fact that the net change in microglial/macrophage cell activation after PPT was less pronounced in transgenic animals than in WT mice, lead as to speculate that IL-6 induced a “primed” microglia/macrophage phenotype. Accordingly, some of the features observed in the population of microglia/macrophages of transgenic mice are commonly linked to a primed state including higher expression of pro-inflammatory mediators as well as MHCII expression [75]. The first descriptions of primed microglia were done in the context of LPS-administration and after IL-1β treatment and identified primed microglia as an altered state that, among other features, presented an exaggerated response to inflammation. However, nowadays, this primed stated has been also described in physiological aging conditions and stress [37, 73] in relation to the development of chronic neurodegenerative diseases [76]. In fact, the concept of primed microglia has been revised and regarded as having an ongoing state of activation that may respond differently to a secondary stimulation [72]. Furthermore, the concept of microglial “innate immune memory” has been recently demonstrated, with an enhancement or suppression of a secondary insult, depending on the type, duration and intensity of the initial stimulus [72].

## Conclusions

**Fig. 6:**
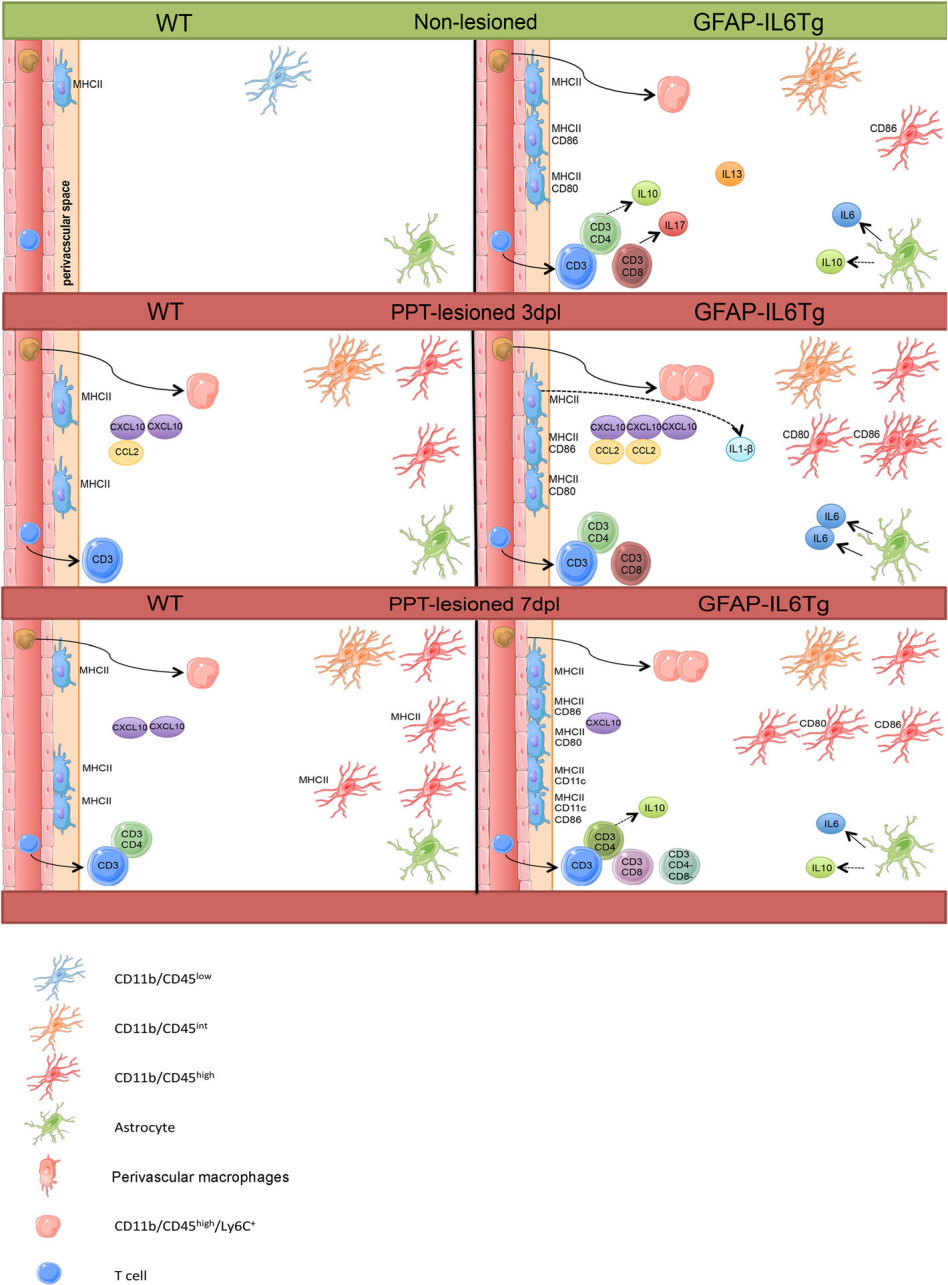
Summary figure. Schematic representation of the main changes observed in the non-lesioned (NL) (green) and PPT-lesioned hippocampus (red) in both WT and GFAP-IL6Tg animals

Altogether, we propose that chronic exposure to IL6 induced an environment characteristic of an innate immune response, with the presence of “primed” microglia/macrophages with a desensitized profile, infiltrating monocytes and T cells as well as increased in IL-6, IL-17, IL10, and IL-13. After PPT, in WT animals, the immune response is characterized by parenchymal microglia showing MHCII and co-stimulatory molecule phenotype. In contrast, the desensitized microglia in transgenic animals is not able to express molecules linked to antigenic presentation following the PPT. This generates a differential microglial phenotype with increased IL-1β that has a negative effect on the pro-regenerative environment and, maybe through action on astrocytes, reduce the axonal sprouting associated with PPT (see Fig. 6).

## Supplementary Information

**Additional file 1: Supplementary Fig. 1.** Area selected for densitometry study. Representative image from WT mice showing Iba1^+^ staining after perforant pathway transection. The selected area (black squares) represented the area analyzed for densitometry. Scale bar = 50μm.

**Additional file 2: Supplementary Fig. 2.** Toluidine blue staining. Representative images, from WT and GFAP-IL6Tg mice, showing toluidine blue staining in the ML of the DG in NL conditions and from 3 to 21 dpl after PPT. Scale bar = 20 μm.

**Additional file 3: Supplementary Fig. 3.** Morphological characterization of microglia. (A–D) Graphs showing the quantification of the area occupied by Iba1^+^ labeled cells (A), (B) the elongation values (value equal to 1 indicates round morphology and high values increased elongation), (C) the sphericity values (value equal to 1 indicates index of sphericity) and (D) the shape factor (high values indicate round shape and low values ramified morphology), calculated for individual cells. The significances are represented as #*p*≤0.001 vs NL of respective group and &*p*≤0.05 vs 3dpl of respective group. Significant differences between genotypes are represented as *p≤0.05 and **p≤0.01.

**Additional file 4: Supplementary Fig. 4.** Microglial cell density. (A) Graph showing the quantification of Pu.1^+^ cells in non-lesioned (NL) and lesioned animals from 2 to 21dpl after PPT, in WT and GFAP-IL6Tg animals. (B) Representative images from WT and GFAP-IL6Tg mice showing Pu.1^+^ staining in the ML of the DG at 14 dpl. Note that transgenic mice showed an increased number of Pu.1^+^ cells in NL and at 14 dpl. Scale bar = 20μm. (C and D) Graphs showing the fold changes increase of Pu.1^+^ cells in WT (C) and GFAP-IL6Tg (D) in comparison to their corresponding NL animals. A minimum of three NL and three lesioned WT and GFAP-IL6Tg at 2, 3, 7, 14 and 21 dpl were analyzed. A total of 6 photographs from 3 different hippocampal sections per animal were used. Data are represented as mean ± SD. The significances are represented as #*p*≤0.01vs NL of respective group; &*p*≤0.05 and %*p*≤0.01 vs 2dpl of respective group and $*p*≤0.001 vs 7dpl of respective group. Significant differences between genotypes are represented as *p≤0.05.

**Additional file 5: Supplementary Fig. 5.** Microglia/macrophages populations. (A) Representative dot plot of CD11b/CD45 expression of cells obtained from the hippocampus of non-lesioned (NL) WT animals. The square delimits the CD11b^+^/CD45^+^ population of cells used in this study. Representative histogram where the populations of CD11b^+^/CD45^low/int^ (microglia) and CD11b^+^/CD45^high^ (macrophages) were defined. (B) Graphs showing the number of cells and the mean fluorescence intensity in the CD11b^+^/CD45^low/int^ and CD11b^+^/CD45^high^ populations in NL and PPT-lesioned animals. Graphs showing the mean fluorescence intensity of CD45 levels in the CD11b^+^/CD45^low/int^ and CD11b^+^/CD45^high^ populations in NL and PPT-lesioned animals. (C) Representative images from WT and GFAP-IL6Tg mice showing CD45 staining in the GL and ML of the DG in NL and PPT-lesioned hippocampus at 3 and 7 dpl. Note that both CD45^+^ ramified (arrowheads) and CD45^+^ round cells (arrows) were observed in both genotypes. Scale bar = 20μm. A minimum of five WT and five GFAP-IL6Tg animals per group were used for this study. Scale bar = 50μm. Data are represented as mean ± SD. The significances are represented as &*p*≤0.01vs NL of respective group; #*p*≤0.05 vs NL of respective group and $*p*≤0.001vs NL of respective group. Significant differences between genotypes are represented as *p≤0.05 and **p≤0.01.

**Additional file 6: Supplementary Fig. 6.** Monocyte infiltration. (A-B) Graphs showing the number of cells expressing the F4/80 and the mean fluorescence. (C-D) Graphs showing the number of cells expressing the monocyte-related marker Ly6C and the mean fluorescence. A minimum of five WT and five GFAP-IL6Tg animals per group were used for this study. Data are represented as mean ± SD. The significances are represented as #*p*≤0.05 vs NL of respective group. Significant differences between genotypes are represented as *p≤0.05, **p≤0.01 and ****p≤0.0001.

**Additional file 7: Supplementary Fig. 7.** Percentages of microglia/macrophage population in NL and after PPT. Representative graph showing the percentage of CD11b^+^/CD45^low/int^ (dark grey) and CD11b^+^/CD45^high^ (light grey) population in both WT and GFAP-IL6Tg animals in NL conditions and after PPT.

**Additional file 8: Supplementary Fig. 8.** CD206 and Laminin expression after PPT. (A) Representative images from WT and GFAP-IL6Tg mice showing CD206 staining in the ML of the DG at 7 dpl. Black arrows indicate CD206^+^ cells. Scale bar = 20μm. (B) Representative images, from WT and GFAP-IL6Tg mice, of double IHC combining MHCII (red) and Laminin (green) at 7 dpl. White arrows indicate MHCII^+^ cells in the perivascular space. Scale bar = 10μm

**Additional file 9: Supplementary Fig. 9.** ICOSL expression. (A) Graph showing the number of ICOSL^+^ cells in non-lesioned (NL) and PPT-lesioned hippocampus, from 3 to 7 dpl, within the CD11b^+^/CD45^low/int^ and CD11b^+^/CD45^high^ cell populations. A minimum of five WT and five GFAP-IL6Tg animals per group were used for this study. Data are represented as mean ± SD. The significances are represented as #*p*≤0.05 vs NL of respective group.

**Additional file 10: Supplementary Fig. 10.** Cytokines and chemokines expression. Graphs showing the time course of expression of IL-2, IL-6, IFNγ, IL1β, IL12p70, IL-17, IL-10, IL-13, IL-9, IL-5, IL-4, CXCL10 and CCL2 in non-lesioned (NL) and PPT-lesioned animals from 3 to 14dpl, in both WT and GFAP-IL6Tg animals. (N) Graph showing the IL-6 levels in serum in both NL WT and NL GFAP-IL6Tg animals. At least four WT and five GFAP-IL6Tg animals for each time point were used. Data are represented as mean ± SD. The significances are represented as #*p*≤0.05, %*p*≤0.01 and &*p*≤0.001 vs NL of respective group; ^*p*≤0.1, $*p*≤0.05, ´´*p*≤0.01 and ∞*p*≤0.001 vs 3dpl of respective group and α*p*≤0.05 vs 7dpl of respective group. Significant differences between genotypes are represented as ***p≤0.001, **p≤0.01, *p≤0.05.

**Additional file 11: Supplementary Fig. 11.** Non-lesioned study in WT and GFAP-IL6Tg animals. Representative graphs showing the differences between WT and GFAP-IL6Tg animals in NL conditions. A minimum of three WT and three GFAP-IL6Tg animals per group were used. Data are represented as mean ± SD. The significances are represented ***p≤0.0001, **p≤0.01, *p≤0.05.

**Additional file 12: Supplementary Fig. 12.** Gating strategy for flow cytometry. (A – F) Representative dot plot and histogram plot from individual hippocampus from WT and GFAP-IL6Tg animals in NL and after PPT. First, population/live cells were gated based on SSC-A and FSC-A, and then microglia/macrophage cells were gated based on CD45 and CD11b expression. CD11b^+^/CD45^low/int^ and CD11b^+^/CD45^high^ population were discriminated according to the levels of CD45. Microglia/macrophages phenotype were studied by MHCII, CD80, CD86, Ly6C and F4/80 expression and gated based on CD11b^+^/CD45^+^/Igs isotype control antibodies.

## Data Availability

All data generated or analyzed during this study are included in this published article [and its supplementary information files].

## Abbreviations

BB: Blocking buffer
BBB: Blood-brain barrier
BrdU: 5′ Bromodeoxyuridine
CNS: Central nervous system
DAB: 3,3-Diaminobenzidine
DAPI: 4,9,6-Diamidino-2-phenylindole
DG: Dentate gyrus
dpl: Day post lesion
GFAP: Glial fibrillary acidic protein
GFAP-IL6Tg: Transgenic mice with astrocyte-targeted IL6-production
GL: Granular layer
IHC: Immunohistochemistry
IL: Interleukin
IML: Inner molecular layer
i.p.: Intraperitoneally
ML: Molecular layer
MML: Medial molecular layer
NL: Non-lesioned
OML: Outer molecular layer
PBS: Phosphate buffer solution
PP: Perforant pathway
PPT: Perforant pathway transection
RT: Room temperature
TBS: Tris-buffered saline
TBST: TBS containing 1% Triton-X100
Tg: Transgenic
WB: Wash buffer
WT: Wild-type
