# Supplementary figures and images for: Chronic exposure to IL-6 induces a desensitized phenotype of the microglia
Source: J Neuroinflammation. 2021 Jan 22;18:31. doi: 10.1186/s12974-020-02063-1 (PMC7821504; doi:10.1186/s12974-020-02063-1)

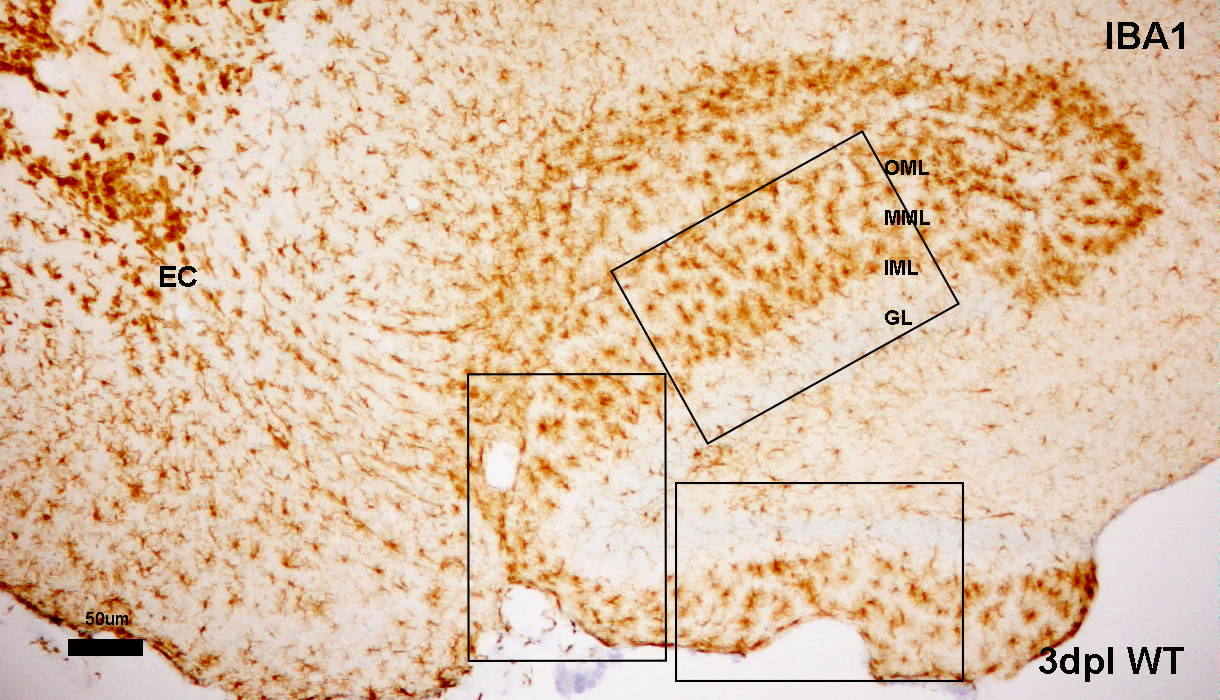

Supplement: Supplementary file 1 — Additional file 1: Supplementary Fig. 1. Area selected for densitometry study. Representative image from WT mice showing Iba1+ staining after perforant pathway transection. The selected area (black squares) represented the area analyzed for densitometry. Scale bar = 50μm. [file 12974_2020_2063_MOESM1_ESM.tif]

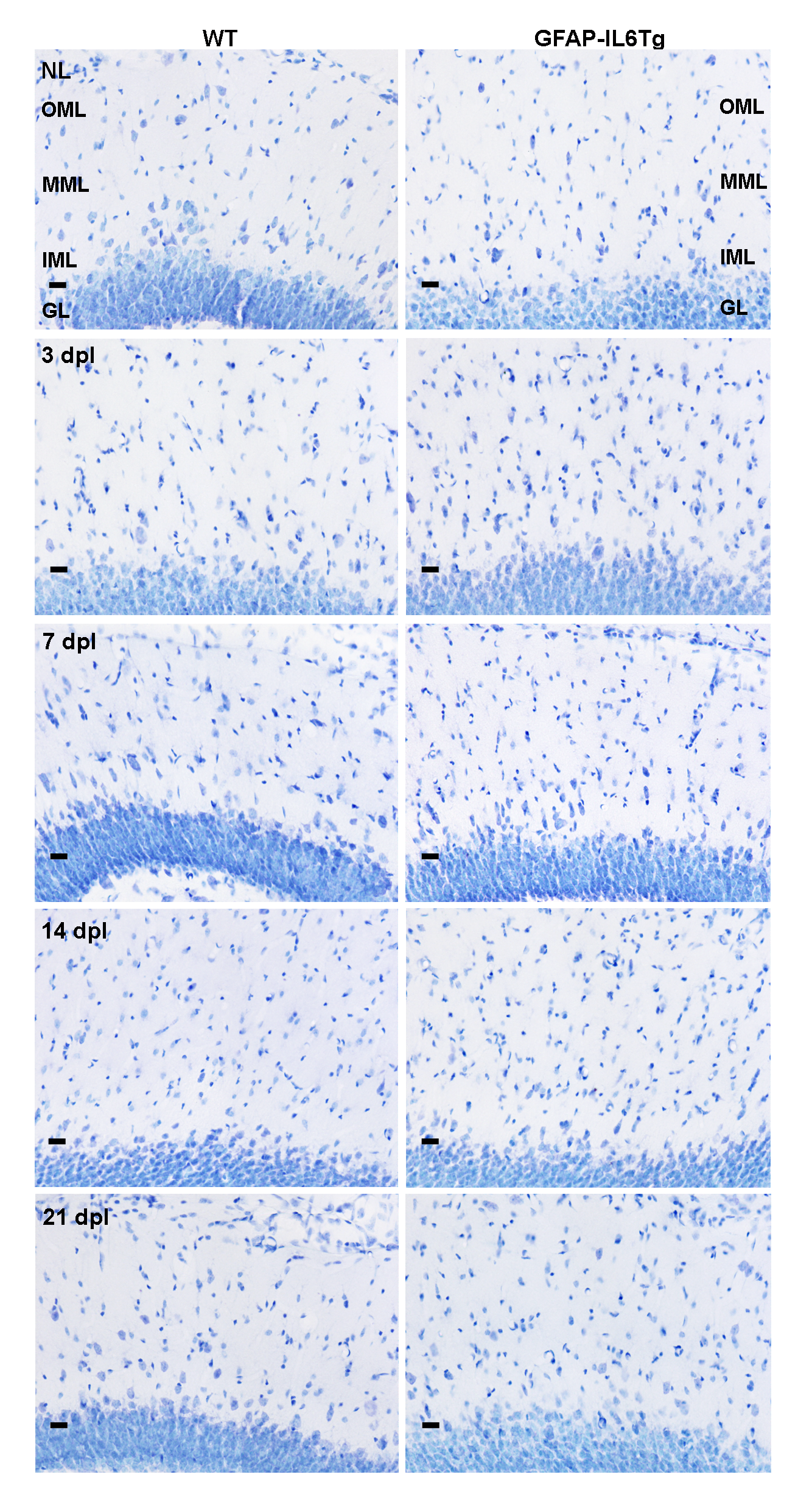

Supplement: Supplementary file 2 — Additional file 2: Supplementary Fig. 2. Toluidine blue staining. Representative images, from WT and GFAP-IL6Tg mice, showing toluidine blue staining in the ML of the DG in NL conditions and from 3 to 21 dpl after PPT. Scale bar = 20 μm. [file 12974_2020_2063_MOESM2_ESM.tif]

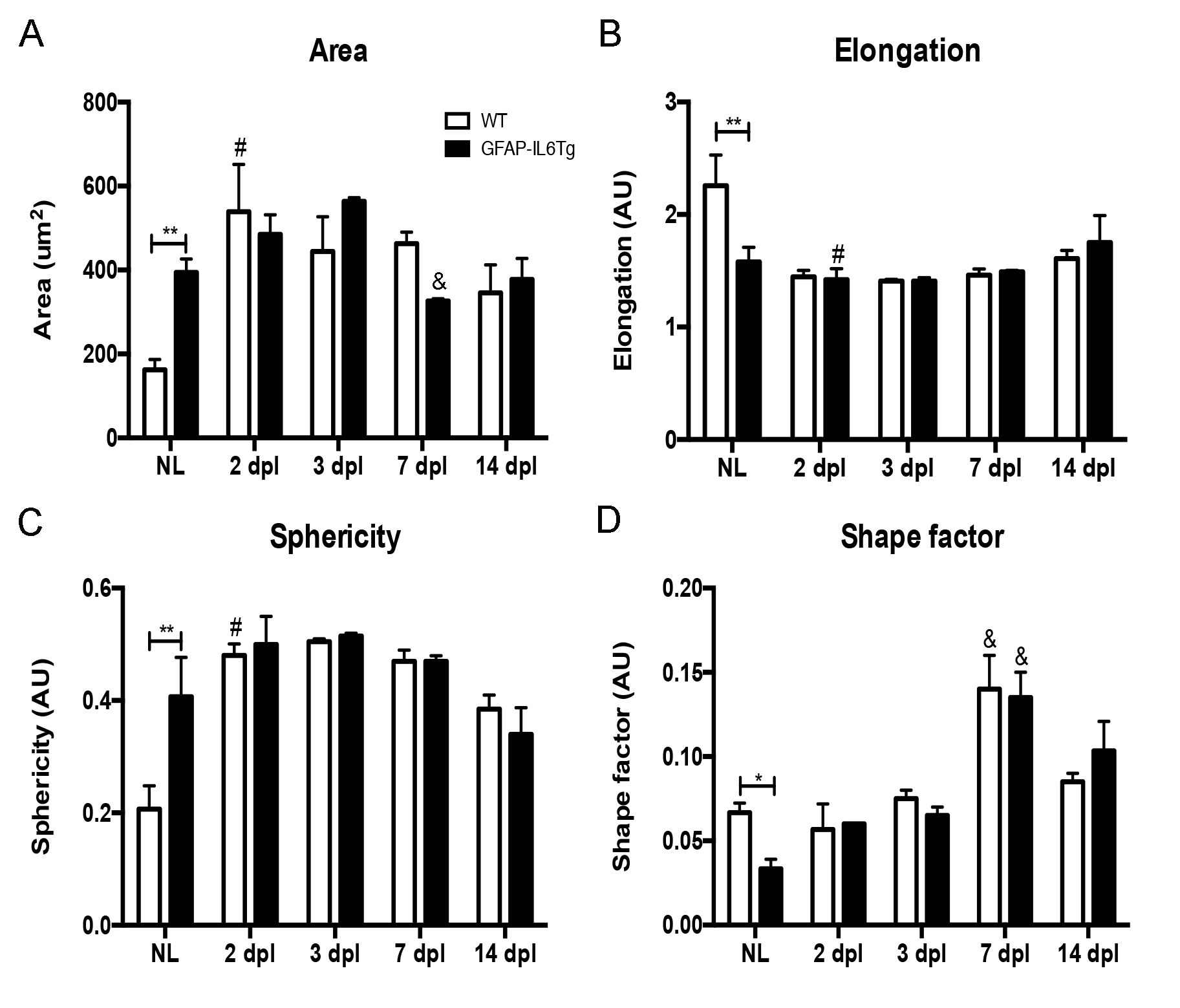

Supplement: Supplementary file 3 — Additional file 3: Supplementary Fig. 3. Morphological characterization of microglia. (A–D) Graphs showing the quantification of the area occupied by Iba1+ labeled cells (A), (B) the elongation values (value equal to 1 indicates round morphology and high values increased elongation), (C) the sphericity values (value equal to 1 indicates index of sphericity) and (D) the shape factor (high values indicate round shape and low values ramified morphology), calculated for individual cells. The significances are represented as #p≤0.001 vs NL of respective group and &p≤0.05 vs 3dpl of respective group. Significant differences between genotypes are represented as *p≤0.05 and **p≤0.01. [file 12974_2020_2063_MOESM3_ESM.tif]

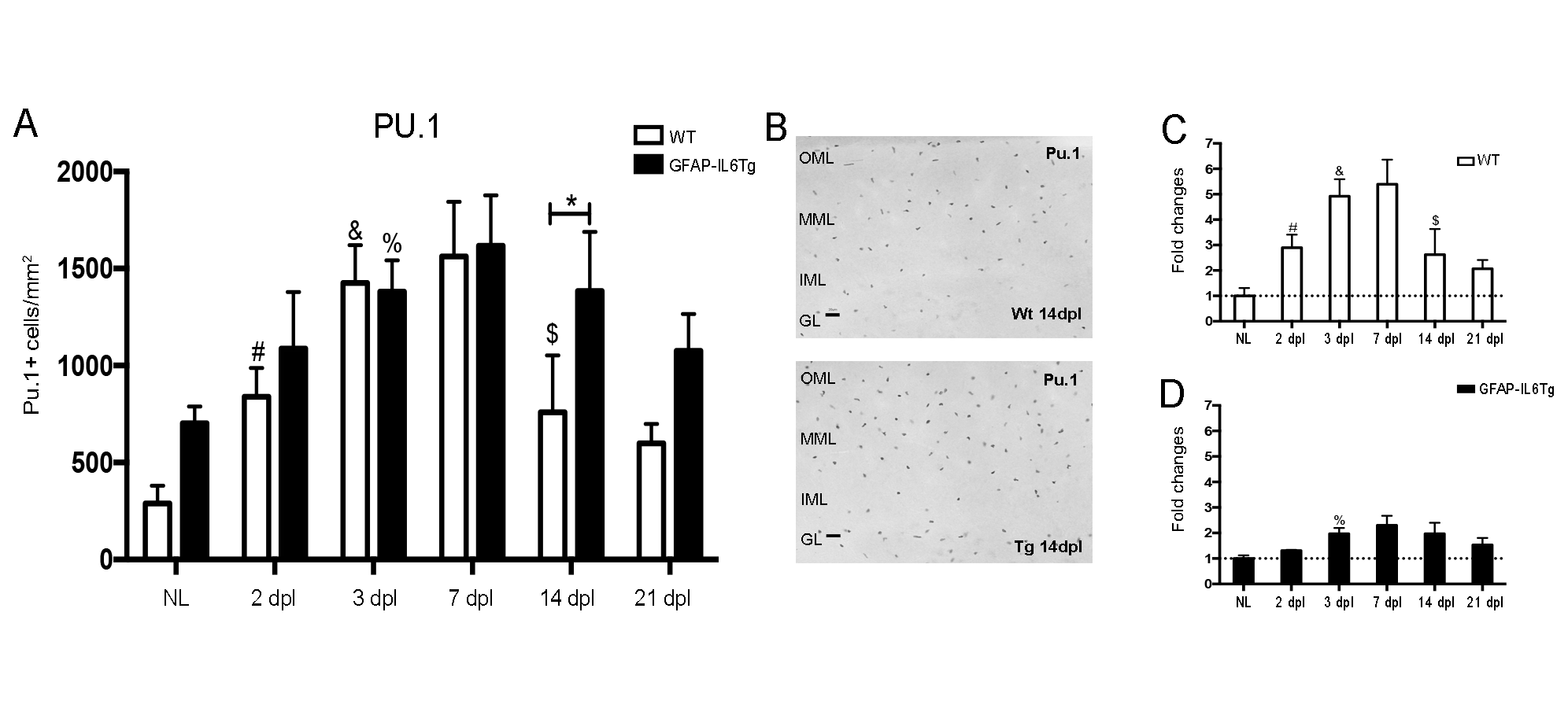

Supplement: Supplementary file 4 — Additional file 4: Supplementary Fig. 4. Microglial cell density. (A) Graph showing the quantification of Pu.1+ cells in non-lesioned (NL) and lesioned animals from 2 to 21dpl after PPT, in WT and GFAP-IL6Tg animals. (B) Representative images from WT and GFAP-IL6Tg mice showing Pu.1+ staining in the ML of the DG at 14 dpl. Note that transgenic mice showed an increased number of Pu.1+ cells in NL and at 14 dpl. Scale bar = 20μm. (C and D) Graphs showing the fold changes increase of Pu.1+ cells in WT (C) and GFAP-IL6Tg (D) in comparison to their corresponding NL animals. A minimum of three NL and three lesioned WT and GFAP-IL6Tg at 2, 3, 7, 14 and 21 dpl were analyzed. A total of 6 photographs from 3 different hippocampal sections per animal were used. Data are represented as mean ± SD. The significances are represented as #p≤0.01vs NL of respective group; &p≤0.05 and %p≤0.01 vs 2dpl of respective group and $p≤0.001 vs 7dpl of respective group. Significant differences between genotypes are represented as *p≤0.05. [file 12974_2020_2063_MOESM4_ESM.tif]

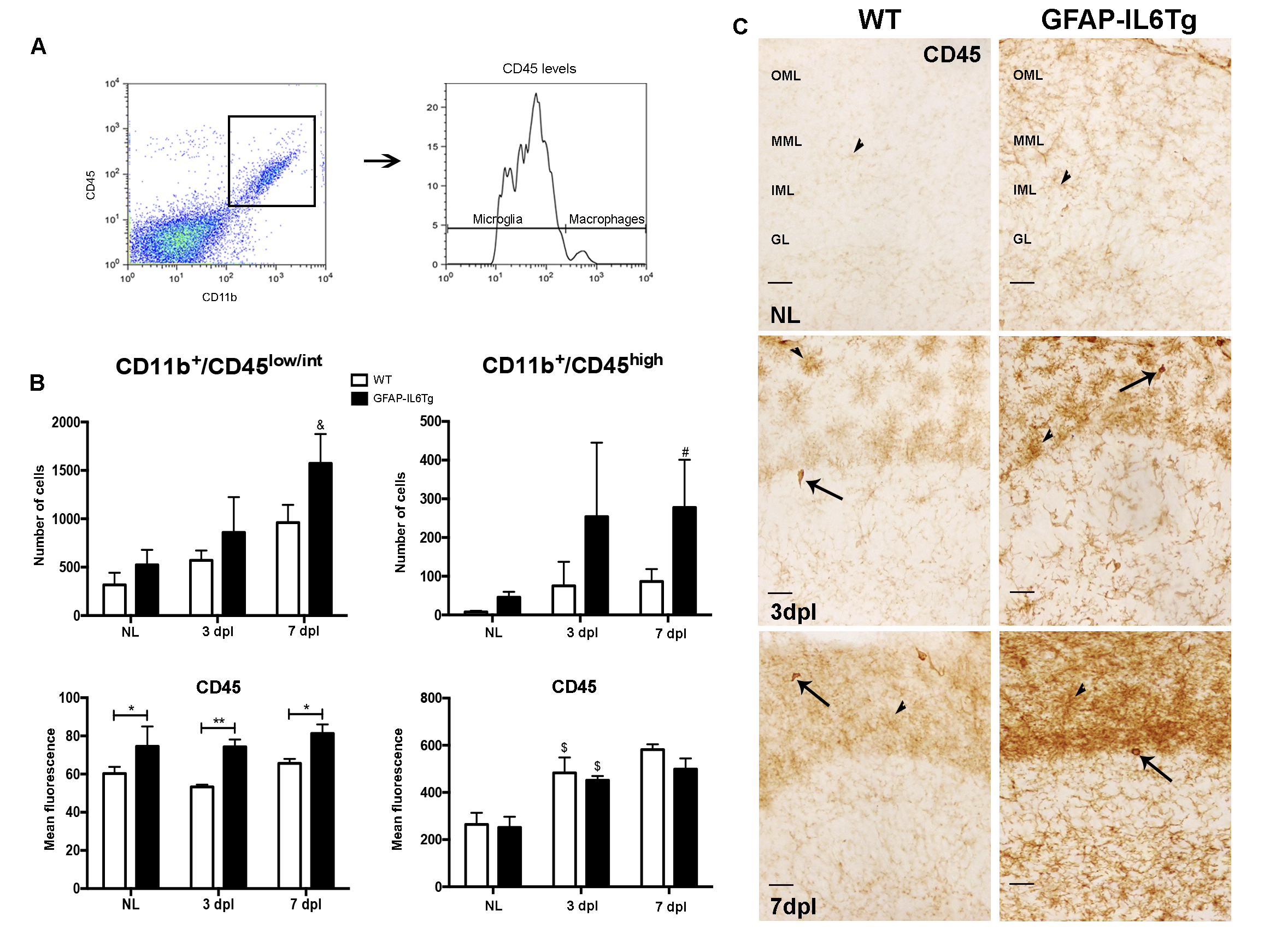

Supplement: Supplementary file 5 — Additional file 5: Supplementary Fig. 5. Microglia/macrophages populations. (A) Representative dot plot of CD11b/CD45 expression of cells obtained from the hippocampus of non-lesioned (NL) WT animals. The square delimits the CD11b+/CD45+ population of cells used in this study. Representative histogram where the populations of CD11b+/CD45low/int (microglia) and CD11b+/CD45high (macrophages) were defined. (B) Graphs showing the number of cells and the mean fluorescence intensity in the CD11b+/CD45low/int and CD11b+/CD45high populations in NL and PPT-lesioned animals. Graphs showing the mean fluorescence intensity of CD45 levels in the CD11b+/CD45low/int and CD11b+/CD45high populations in NL and PPT-lesioned animals. (C) Representative images from WT and GFAP-IL6Tg mice showing CD45 staining in the GL and ML of the DG in NL and PPT-lesioned hippocampus at 3 and 7 dpl. Note that both CD45+ ramified (arrowheads) and CD45+ round cells (arrows) were observed in both genotypes. Scale bar = 20μm. A minimum of five WT and five GFAP-IL6Tg animals per group were used for this study. Scale bar = 50μm. Data are represented as mean ± SD. The significances are represented as &p≤0.01vs NL of respective group; #p≤0.05 vs NL of respective group and $p≤0.001vs NL of respective group. Significant differences between genotypes are represented as *p≤0.05 and **p≤0.01. [file 12974_2020_2063_MOESM5_ESM.tif]

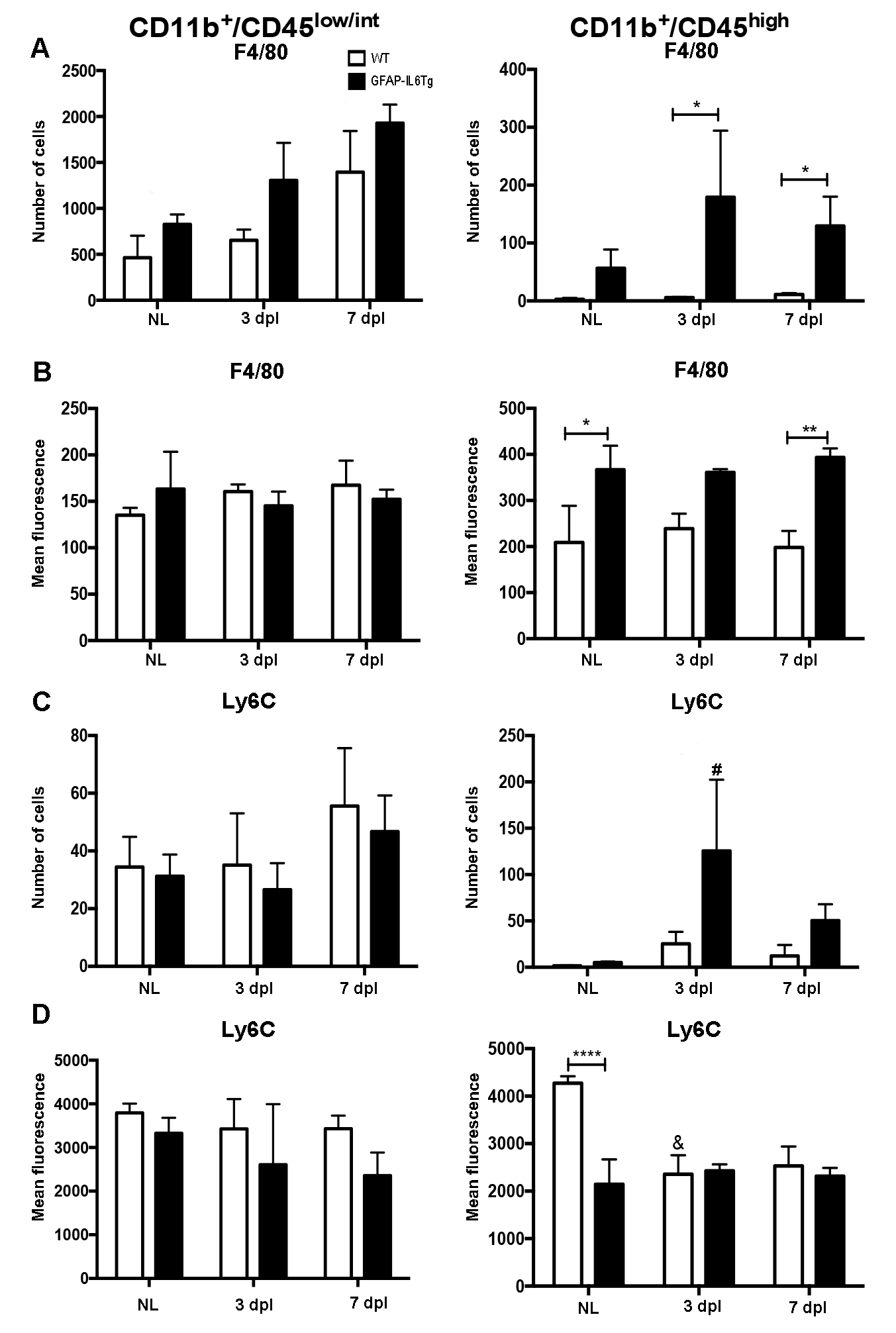

Supplement: Supplementary file 6 — Additional file 6: Supplementary Fig. 6. Monocyte infiltration. (A-B) Graphs showing the number of cells expressing the F4/80 and the mean fluorescence. (C-D) Graphs showing the number of cells expressing the monocyte-related marker Ly6C and the mean fluorescence. A minimum of five WT and five GFAP-IL6Tg animals per group were used for this study. Data are represented as mean ± SD. The significances are represented as #p≤0.05 vs NL of respective group. Significant differences between genotypes are represented as *p≤0.05, **p≤0.01 and ****p≤0.0001. [file 12974_2020_2063_MOESM6_ESM.tif]

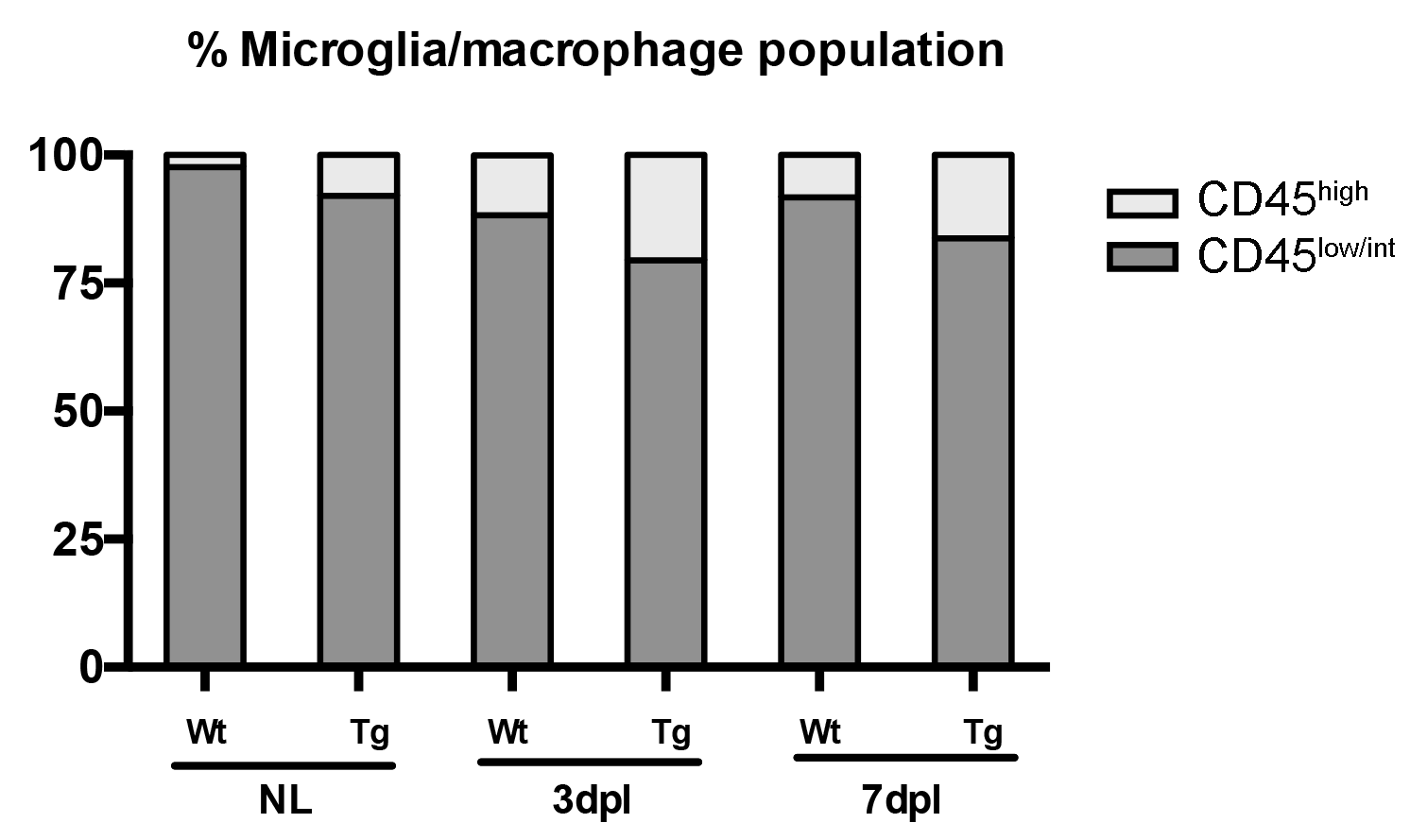

Supplement: Supplementary file 7 — Additional file 7: Supplementary Fig. 7. Percentages of microglia/macrophage population in NL and after PPT. Representative graph showing the percentage of CD11b+/CD45low/int (dark grey) and CD11b+/CD45high (light grey) population in both WT and GFAP-IL6Tg animals in NL conditions and after PPT. [file 12974_2020_2063_MOESM7_ESM.tif]

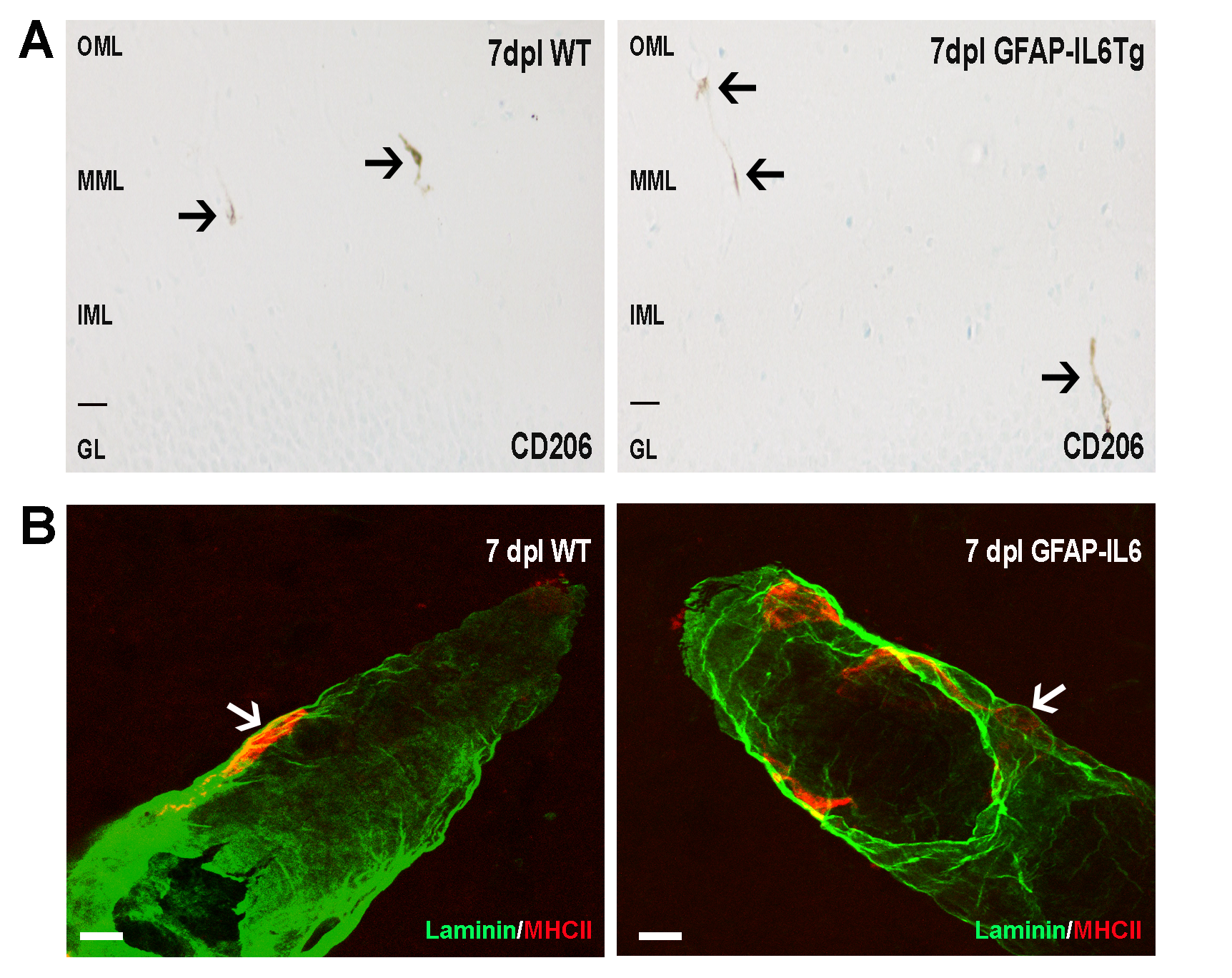

Supplement: Supplementary file 8 — Additional file 8: Supplementary Fig. 8. CD206 and Laminin expression after PPT. (A) Representative images from WT and GFAP-IL6Tg mice showing CD206 staining in the ML of the DG at 7 dpl. Black arrows indicate CD206+ cells. Scale bar = 20μm. (B) Representative images, from WT and GFAP-IL6Tg mice, of double IHC combining MHCII (red) and Laminin (green) at 7 dpl. White arrows indicate MHCII+ cells in the perivascular space. Scale bar = 10μm [file 12974_2020_2063_MOESM8_ESM.tif]

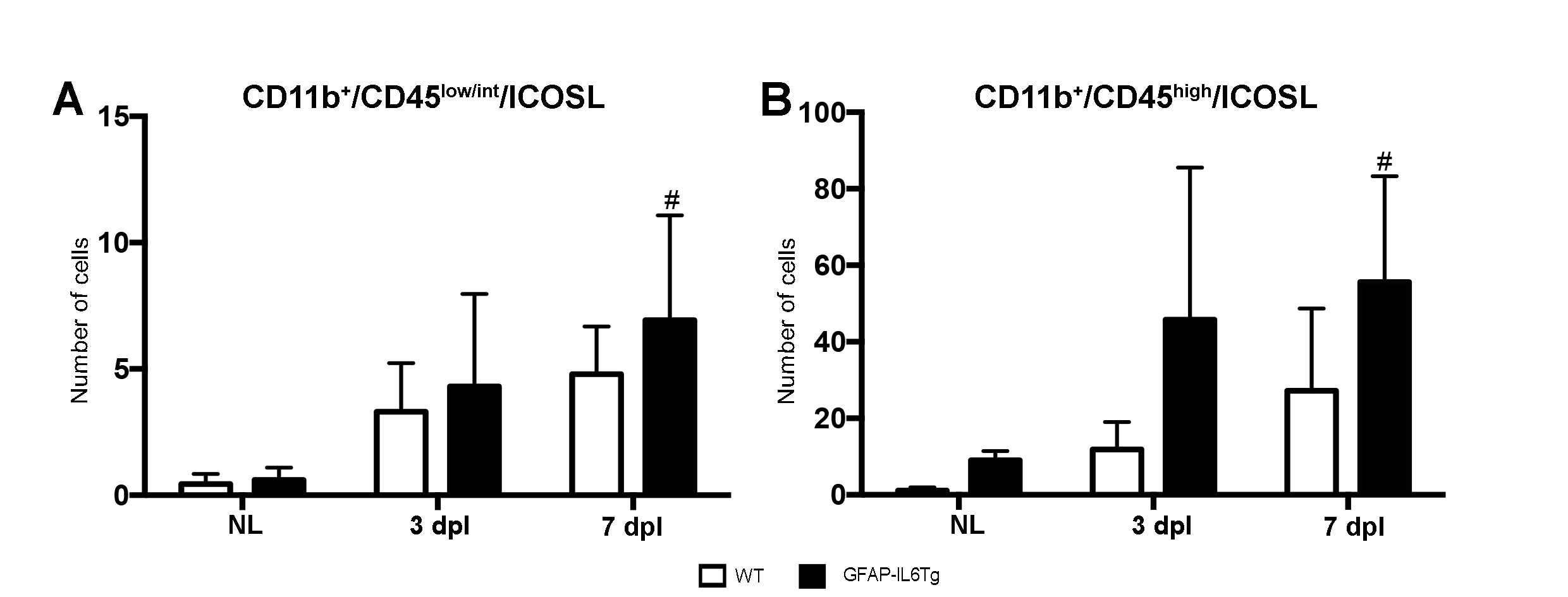

Supplement: Supplementary file 9 — Additional file 9: Supplementary Fig. 9. ICOSL expression. (A) Graph showing the number of ICOSL+ cells in non-lesioned (NL) and PPT-lesioned hippocampus, from 3 to 7 dpl, within the CD11b+/CD45low/int and CD11b+/CD45high cell populations. A minimum of five WT and five GFAP-IL6Tg animals per group were used for this study. Data are represented as mean ± SD. The significances are represented as #p≤0.05 vs NL of respective group. [file 12974_2020_2063_MOESM9_ESM.tif]

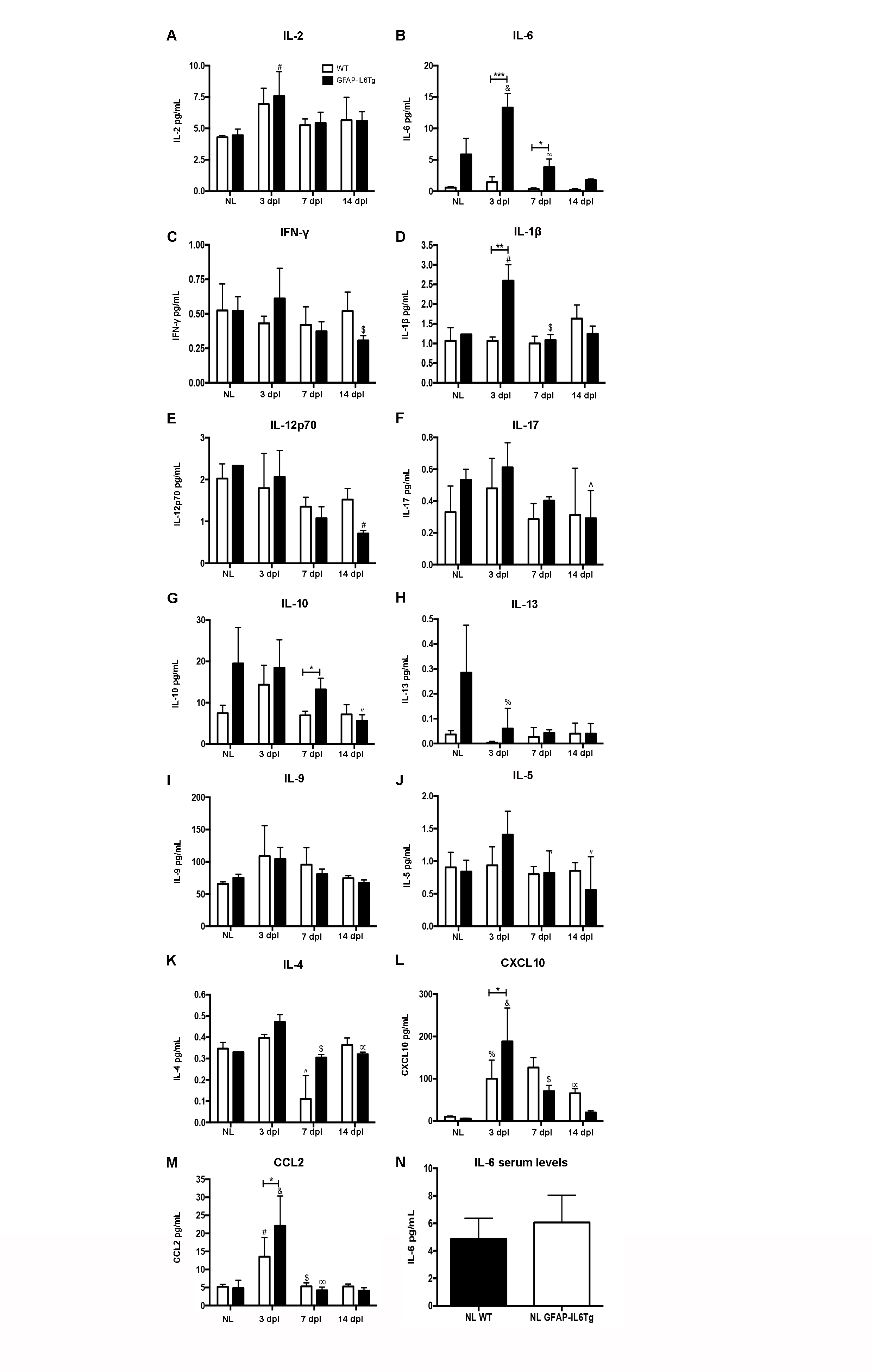

Supplement: Supplementary file 10 — Additional file 10: Supplementary Fig. 10. Cytokines and chemokines expression. Graphs showing the time course of expression of IL-2, IL-6, IFNγ, IL1β, IL12p70, IL-17, IL-10, IL-13, IL-9, IL-5, IL-4, CXCL10 and CCL2 in non-lesioned (NL) and PPT-lesioned animals from 3 to 14dpl, in both WT and GFAP-IL6Tg animals. (N) Graph showing the IL-6 levels in serum in both NL WT and NL GFAP-IL6Tg animals. At least four WT and five GFAP-IL6Tg animals for each time point were used. Data are represented as mean ± SD. The significances are represented as #p≤0.05, %p≤0.01 and &p≤0.001 vs NL of respective group; ^p≤0.1, $p≤0.05, ´´p≤0.01 and ∞p≤0.001 vs 3dpl of respective group and αp≤0.05 vs 7dpl of respective group. Significant differences between genotypes are represented as ***p≤0.001, **p≤0.01, *p≤0.05. [file 12974_2020_2063_MOESM10_ESM.tif]

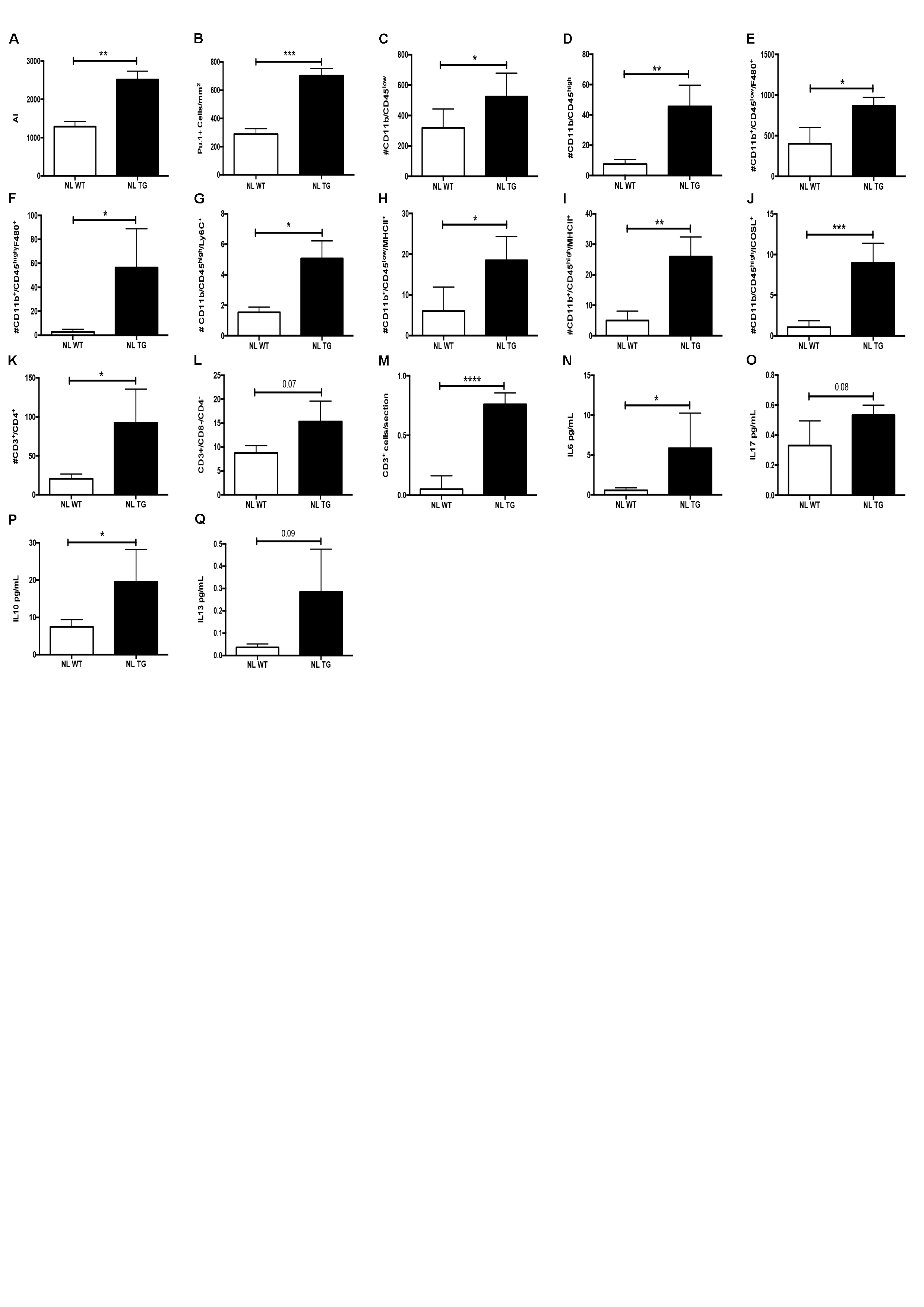

Supplement: Supplementary file 11 — Additional file 11: Supplementary Fig. 11. Non-lesioned study in WT and GFAP-IL6Tg animals. Representative graphs showing the differences between WT and GFAP-IL6Tg animals in NL conditions. A minimum of three WT and three GFAP-IL6Tg animals per group were used. Data are represented as mean ± SD. The significances are represented ***p≤0.0001, **p≤0.01, *p≤0.05. [file 12974_2020_2063_MOESM11_ESM.tif]

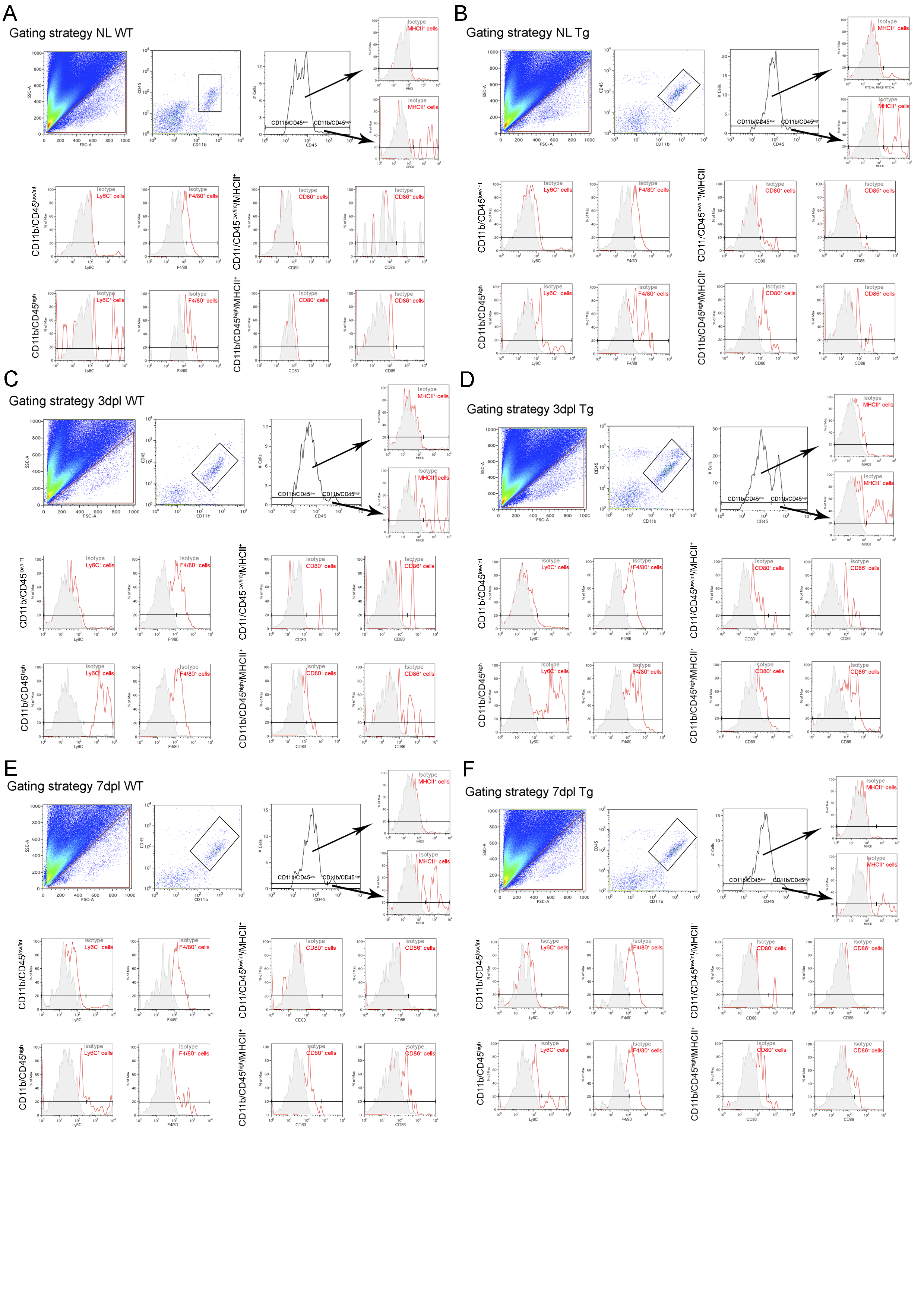

Supplement: Supplementary file 12 — Additional file 12: Supplementary Fig. 12. Gating strategy for flow cytometry. (A – F) Representative dot plot and histogram plot from individual hippocampus from WT and GFAP-IL6Tg animals in NL and after PPT. First, population/live cells were gated based on SSC-A and FSC-A, and then microglia/macrophage cells were gated based on CD45 and CD11b expression. CD11b+/CD45low/int and CD11b+/CD45high population were discriminated according to the levels of CD45. Microglia/macrophages phenotype were studied by MHCII, CD80, CD86, Ly6C and F4/80 expression and gated based on CD11b+/CD45+/Igs isotype control antibodies. [file 12974_2020_2063_MOESM12_ESM.tif]
